# A novel role of secreted methionine adenosyltransferase α2 in colorectal liver metastases

**DOI:** 10.1186/s13046-025-03599-x

**Published:** 2025-12-02

**Authors:** Monica Justo, Youngyi Lim, Heping Yang, Andrea Floris, Swati Chandla, Manisha Dagar, Alexandra Gangi, Edwin Posadas, Mouad Edderkaoui, Stephen Pandol, Neil Bhowmick, Maria Lauda Tomasi, Shelly C. Lu

**Affiliations:** 1 Jim and Eleanor Randall Department of Surgery, Cedars-Sinai Medical Center, Los Angeles, CA 90048 USA; 2 Department of Medicine, Karsh Division of Gastroenterology and Hepatology, Cedars-Sinai Medical Center, Davis Building, Room #2093, 8700 Beverly Blvd.,, Los Angeles, CA 90048 USA; 3 Division of Oncology, Los Angeles, USA; 4 Department of Biomedical Sciences, Los Angeles, USA; 5 Cancer Institute, Cedars-Sinai Medical Center, Los Angeles, CA 90048 USA

**Keywords:** Colorectal cancer, Colorectal liver metastasis, Extracellular vesicles, Exosomes, FAK, Hepatocytes, MAT1A, MAT2A

## Abstract

**Background:**

Colorectal liver metastasis (CRLM) occurs frequently in patients with colorectal cancer (CRC). Methionine adenosyltransferase (MAT) catalyzes the formation of S-adenosylmethionine, the principal methyl donor. *MAT1A* (encodes MATα1) is expressed mainly in normal adult liver, whereas *MAT2A* (encodes MATα2) is expressed in all extrahepatic tissues. MAT1A is a major defense against CRLM as loss of *Mat1a* sensitizes the liver to CRLM. In contrast, MAT2A is overexpressed in CRC and promotes oncogenicity. Here, we sought to determine if CRCs secrete MATα2 and if this influences CRLM.

**Methods:**

Our study included human hepatocytes, human CRC cells, extracellular vesicle (EV) isolation, chromatin immunoprecipitation (ChIP), ChIP-seq, promoter activity assays, proliferation, migration, and invasion assays, western blotting, immunohistochemistry and immunofluorescence. We confirmed some of the findings using human hepatocyte spheroids, CRLM and normal liver tissue array, and plasma samples.

**Results:**

CRCs secrete MATα2 in free but truncated form (MATα2-t) and intact within EVs (EV-MATα2). EV-MATα2 can be internalized by human hepatocytes and CRCs, found within the nucleus, which then binds to *MAT1A* and *MAT2A* promoters on ChIP to lower and increase *MAT1A* and *MAT2A* promoter activities, respectively. In human CRLM samples, hepatocytes in nontumor regions express lower MATα1 but higher MATα2 as compared to normal liver. Treating RKO cells with EVs released from RKO cells overexpressing MAT2A promoted cell proliferation, migration, and invasion. MATα2-t was detected at a higher level in media from colon, pancreatic, and prostate cancer cell lines than corresponding normal epithelial cells as well as in the plasma of CRC patients as compared to healthy controls. RKO cells treated with MATα2-t activated focal adhesion kinase (FAK), an important kinase for cancer cell evasion of apoptosis. Conversely, treatment with MATα2 neutralizing antibody inhibited FAK and induced apoptosis.

**Conclusions:**

CRC cells secrete both MATα2 within EVs and free MATα2-t. EV-MATα2 can be internalized and act as a transcription factor to lower hepatocytes’ MAT1A, the major defense against CRLM, while promoting CRC oncogenicity. Freely released MATα2-t acts as a ligand in an autocrine fashion to activate FAK, which is essential for CRC survival. Taken together, secreted MATα2 plays an essential role in promoting CRLM.

**Supplementary Information:**

The online version contains supplementary material available at 10.1186/s13046-025-03599-x.

## Background

Colorectal cancer (CRC) is the second most common cancer worldwide, and the third leading cause of cancer-related mortality in the United States [1, 2]. The liver is the most frequent site of CRC metastasis, which is the major contributor to poor prognosis and death [3]. Up to 50% of patients develop colorectal liver metastases (CRLM) [4]. Currently, surgical resection remains the only curative treatment for CRLM, but only 20% of patients initially qualify for complete resection [5]. Even after surgical resection, recurrence rates remain high, with a 60–70% relapse rate within three years and a 5-year survival rate of 40–50% [5–7]. While systemic chemotherapy and liver-directed therapies like ablation or radiation down-grade the initially unresectable metastasis into resectable disease, survival outcomes remain poor [4, 8]. Thus, understanding the molecular mechanisms underlying CRC’s ability to seed the liver is crucial for identifying new therapeutic targets to prevent or mitigate metastasis progression. A critical regulator of liver health is methionine adenosyltransferase (MAT), which catalyzes the synthesis of S-adenosylmethionine (SAMe), the principal methyl donor and precursor of glutathione in the liver [9]. MAT isoforms are encoded by MAT1A (encodes for MATα1) and MAT2A (encodes for MATα2), with MAT1A predominantly expressed in hepatocytes and cholangiocytes of the healthy adult liver and MAT2A in all extrahepatic tissues and non-parenchymal cells of the liver [10, 11]. Elevated MAT2A expression occurs in multiple cancers and has been considered as a negative prognostic indicator, including in CRC [12–18]. We reported that loss of *Mat1a* sensitized the liver to CRLM, suggesting MAT1A is a major defense against CRLM [19]. We found MAT1A expression is downregulated in human hepatocytes when cultured with CRC cells suggesting that CRC-derived factors may modulate the hepatic level of MAT1A [19]. Here, we examined if CRCs secrete MATα2 and if this plays a role in CRLM. Our study revealed CRC cells secrete two forms of MATα2, the full-length form that is within extracellular vesicles (EVs), and a novel truncated form (MATα2-t) that is free. EV-MATα2 can be internalized by hepatocytes and CRCs and act as a transcription factor to lower MAT1A and raise MAT2A transcription, whereas MATα2-t can act as a ligand to activate focal adhesion kinase (FAK) in CRCs. Neutralizing MATα2-t with anti-MATα2 antibody inhibited FAK and induced apoptosis. These findings reveal a previously unknown role of the secreted MATα2 in promoting CRLM.

## Methods

### Cell culture

RKO, HT29, MIA PaCa-2, 22Rv1, and RWPE-1 cells were purchased from American Type Culture Collection (cat. CRL-2577, HTB-38, CRL-1420, CRL-2505, CRL-3607; Rockville, MD). Cells were grown following protocol provided by American Type Culture Collection. Briefly, RKO cells were cultured with MEM media (cat. MT10010CV; Corning, Tewksbury, MA), 10% fetal bovine serum (FBS), 1% penicillin-streptomycin solution (Cytiva, Marlborough, MA), 1% sodium pyruvate, 1% MEM nonessential amino acids (cat. 11140-50; Grand Island Biological Company, Carlsbad, CA). MIA PaCa-2 and HT29 cells were cultured with DMEM media (cat. MT10-013-CV; Corning, Tewksbury, MA), 10% FBS, 1% penicillin-streptomycin solution. 22Rv1 cells were cultured with RPMI 1640 media (cat.10–040-CV; Corning, Tewksbury, MA), 10% FBS, 1% penicillin-streptomycin solution. RWPE-1 cells were cultured with keratinocyte serum free medium (cat.10744019; ThermoFisher, Carlsbad, CA), 10% FBS, 1% penicillin-streptomycin. HCoEpC cells were purchased from iXCells Biotechnologies (cat. 10HU-096; San Diego, CA) and cultured with Epithelial Cell Growth Medium (cat. MD-0041; iXCells Biotechnologies, San Diego, CA). HPDE cells were kindly provided by Dr. Stephen Pandol (Cedars-Sinai Medical Center, CA) and cultured with keratinocyte serum free medium,10% FBS, 1% penicillin-streptomycin solution.

Cryopreserved human hepatocytes were purchased from Grand Island Biological Company (Lot# HU8191, Carlsbad, CA). Human hepatocytes were thawed from cryovials and cleaned using 90% Percoll solution per Emulate Liver-Chip Tri-Culture protocol (Boston, MA). Hepatocytes (0.5 × 10^6^) were subsequently plated on collagen-coated 6-well plates utilizing 1 cryo-vial/2 × 6-well plates or 1 cryo-vial/2 × 10-cm dishes. Human hepatocytes were cultured with DMEM media, 10% FBS, 1% penicillin-streptomycin solution. After hepatocyte attachment (approximately 3.5 h), floating cells were removed by rinsing with culture media. After replacement of media, hepatocytes were treated for 24 h with 50uL or 250uL, respectively, of isolated exosomes as described below.

Human liver spheroids were cultured and maintained following Star protocol [20]. Briefly, human hepatocytes (5 × 10^6^) were thawed and suspended in a gradient for centrifugation containing 13.5mL Percoll, 1.5mL 10X Dulbecco’s Phosphate Buffered Saline (DPBS), and 35mL of complete medium and centrifuged 100 xg for 10 min at room temperature. Per the protocol, complete medium was created with 42.65mL DMEM media, 1.25mL (4-(2-Hydroxyethyl)piperazine-1-ethanesulfonic acid) (HEPES) (cat. 15630080; Thermo Fisher, Carlsbad, CA), 0.5mL penicillin-streptomycin solution, 5mL FBS, 0.5mL Insulin-Transferrin-Selenium (ITS-G) (cat. 41400045; ThermoFisher, Carlsbad, CA). The supernatant was gently removed and the pellet was resuspended with 50mL complete medium. Cells were centrifuged 100 xg for 5 min at room temperature. The pellet was then resuspended in 10mL complete medium and 100uL of the cell suspension was added to each well of 96-well ULA plate (cat. CLS3474-24EA; Corning, Tewksbury, MA). The plate was centrifuged for 5 min at 100 xg at room temperature. Then, the plate was incubated at 37 °C, 5% CO_2_ while shaking at 300 rpm for 25 min. Following shaking, the plate was centrifuged at 100 xg for 5 min at room temperature. Then 100uL of growth medium was added to each well, careful to not disturb the cells at the bottom of the well. The growth medium was created by adding 5mL of FBS to 45 mL complete medium. The plate was again incubated at 37 °C, 5% CO_2_ while shaking at 300 rpm for 25 min. Then, the plate was incubated at 37 °C, 5% CO_2_ for 7 days. At day 7, liver spheroids were treated with 5uL per well of isolated exosomes as described below.

### In vitro transient transfection

MAT2A overexpression vectors in DDK(N-terminal)-His(C-terminal) and His-tag (C-terminal) were purchased from Origene (Rockville, MD). Plasmid was isolated using ZymoPURE Plasmid Maxiprep Kit (cat. D4203; Zymo Research, Irvine, CA) per manufacturer’s protocol. RKO cells were cultured on 6-well plates (0.3 × 10^6^ cells/well) or 10-cm/dishes (2 × 10^6^ cells/dish) and transfected with 4uL/well or 20uL/dish of JetPrime transfection Reagent (cat. 101000046; Polyplus, New York, NY). 2ug of plasmid was added per well or 10ug per 10-cm/dish. After 4 h of incubation, the culture medium was changed. After 48 h of overexpression, cells underwent whole protein isolation for western blot analysis and culture media was collected for western blot analysis or utilized for exosome isolation.

The same JetPrime transfection reagent and protocol (Polyplus, New York, NY) was utilized for transfection of promoter assay plasmids.

### Exosome isolation

RKO and HT29 cells were plated 2 × 10^6^ cells per 10-cm/dish. Cells were transiently transfected to overexpress MAT2A in His-tag or empty vector control as above. After 48 h of overexpression, culture media was collected and cleaned via centrifugation at 2000 x g for 30 min. Exosomes were isolated from the culture media using Total Exosome Isolation Kit (from cell culture media) (cat. 4478359; Thermo Fisher Scientific, Burlington, MA). Exosomal pellets were precipitated via centrifugation in 1.5mL aliquots. Pellets were subsequently dissolved in 40uL PBS/aliquot and combined.

### Nanosight analysis

Nanosight analysis was performed by System Biosciences (Palo Alto, CA) to show the effect of MAT2A overexpression in His-tag on exosome secretion. Briefly, RKO cell culture media was prepared as described in exosome isolation. The cell culture media was centrifuged 3000 x g for 15 min and frozen in −80˚C. The frozen media was subsequently processed for Nanosight Analysis.

### Cellular fractionation

For whole-cell protein extraction from RKO cells and primary human hepatocytes, cells were lysed using radioimmunoprecipitation assay buffer containing protease inhibitor (100:1) then underwent centrifugation at 12,000 x g for 10 min at 4˚C. Supernatant was subsequently used for western blot analysis or frozen for further use.

To extract the cytosolic and nuclear fractions from primary human hepatocytes and RKO cells, the Plasma Membrane Protein Isolation and Cell Fractionation Kit (SM-005, Invent Biotechnologies, Plymouth, MN) was used following manufacturer’s protocol.

### Western blot analysis

Protein levels were analyzed by western blot from cell lysates, conditioned media, exosome and human plasma. Specifically, protein cell lysates were prepared using RIPA buffer (cat. 20–188; Millipore Sigma, Burlington, MA), supplemented with protease and phosphatase inhibitors (1:100; cat. 78442; ThermoFisher, Waltham, MA) and quantified by the Bradford protein assay (cat. 5000006; Biorad, Hercules, CA) after centrifugation. For human plasma, samples were diluted in RIPA buffer and normalized to protein content prior to gel loading. Conditioned media from cancer and control cell lines was similarly processed and probed for secreted proteins. After electrophoresis in a 10% SDS-PAGE gel, proteins were transferred to PVDF membranes and blocked in 5% skin milk (Apex, 20–241) for 1 h and then incubated with primary antibody (Supplemental Table 1) at 4 °C overnight. The membranes were then incubated with horseradish peroxidase (HRP)-conjugated antibody (Supplemental Table 1) for 1.5 h. Immunoreactive bands were detected with Radiance ECL kit (cat. AC2204; Azure biosystems, Dublin, CA) or ECL Select Western Blotting Detection Reagent (cat. RPN2235; Cytiva, Marlborough, MA) according to the manufacturer’s instructions. All blots were analyzed by ImageJ software (versions 1.51 and 1.54j) for quantification.

### RNA extraction and real-time polymerase chain reaction (PCR) analysis

Total RNA was isolated from RKO cells, nd primary human hepatocytes and liver spheroids utilizing the Quick-RNA MiniPrep Kit (cat. R1055; Zymo Research, Tustin, CA). RNA subsequently underwent reverse transcription using NxGen M-MuLV Reverse Transcriptase (LGC Biosearch Technologies, Novato, CA). 2uL of reverse transcription product underwent quantitative real-time PCR analysis. *MAT1A*, *MAT2A and FAK* probes were purchased from ThermoFisher (Waltham, MA). *HRPT1* (cat.Hs99999909_m1; ThermoFisher, Waltham, MA) was used as a housekeeping gene (Supplemental Table 2). The delta Ct (ΔCt) obtained was used to find the relative expression of genes according to the following formula: relative expression = 2 − ΔΔCt, where ΔΔCt = ΔCt of respective genes in experimental groups − ΔCt of the same genes in control group.

### Chromatin immunoprecipitation (ChIP)

ChIP assay was completed using Zymo-Spin ChIP Kit (cat. D5210-A; Zymo Research, Tustin, CA). RKO or HT29 cells and primary human hepatocytes were treated with isolated exosomes from RKO or HT29 cells overexpressing MAT2A in His-tag or empty vector as previously described. 3ug of His-Tag (cat. ab213204; Abcam, Cambridge, UK) or POL II (sc-13583; Santa Cruz, Dallas, TX) antibody was used to precipitate chromatin. *MAT1A* and *MAT2A* promoter regions were amplified by PCR using the specific primers indicated in Supplemental Table 2. All PCR products were run on 2% agarose gel and stained with Safe DNA Gel Stain (cat. SAFE01-01; Bioland Scientific, Paramount CA). PCR conditions consisted of 3 min denaturation at 95˚C followed by 35 cycles at 95˚C for 30 s, annealing at 58˚C for 1 min and extension 72˚C for 1 min using Apex 2x Taq RED Master Mix (cat. 42–138; Genesee Scientific, San Diego, CA). Images were captured using Azure 400 Fluorescent Imager (Azure Biosystems, Dublin, CA). Densitometry was analyzed using ImageJ software.

### ChIP-seq analysis

Purified DNA from RKO cells was used to generate sequencing libraries by University of Southern California (USC) Molecular Genomic Core. Libraries were quantified, pooled, and sequenced on an Illumina platform to generate 50-bp single-end reads. Sequencing reads were aligned to the human reference genome (hg19) using Bowtie2 with default parameters. Duplicates were removed, and peak calling was performed using MACS2 with input DNA as control. Significant peaks were identified with a q-value cutoff of 0.05. Peaks were annotated relative to genomic features (promoters, exons, introns, intergenic regions) using HOMER and ChIPseeker.

De novo motifs discovery within MATα2 peaks was performed using RSAT software [21]. Genes associated with MATα2 peaks were subjected to pathway enrichment analysis using the DAVID Bioinformatics Resources and clusterProfiler in R. Enriched pathways were identified based on Gene Ontology (GO) terms and KEGG pathways, with a significance threshold of adjusted *p* < 0.05. Signal tracks of MATα2 ChIP-seq data were converted to bigWig format and visualized using the UCSC Genome Browser. Peaks and binding profiles across genomic loci were displayed alongside RefSeq gene annotations.

### Promoter assay

Primary human hepatocytes were plated at 50–60% confluence on 6-well plates and transfected with human *MAT2A* (−939/+60) and *MAT1A* (−839/+30) promoters that were previously characterized [22, 23] using JetPrime transfection reagent (cat. 101000015; Polyplus, New York, NY). After 4 h of incubation, the cell culture medium was changed, and cells were treated with 50uL of isolated exosomes. After 24 h of exosome treatment, the cells were lysed and read for luminescence using CLARIOstar Plus plate reader (Ortenberg, Germany).

### Immunofluorescence microscopy

RKO cells and primary hepatocytes were grown on coverslips and treated with exosomes for 24 h. The cells were fixed with 10% neutral buffered formalin for 15 min at room temperature. After washing the cells 2 times with PBS, they were permeabilized with PBS containing 0.1% Triton X-100 for 10 min. Cells were washed 3 times with PBS and blocked with 1% Bovine Serum Albumin prepared in PBS containing 0.1% Triton X-100. Then, cells were incubated with 1:200 diluted primary antibodies, DDK (cat. TA50011; Origene, Rockville, MD) or His-Tag (cat. 66005-1-AP; Proteintech, Rosemont, IL) overnight in a humidified chamber at 4˚C. Cells were washed with PBS and subsequently incubated with diluted secondary antibodies (1:200), mouse Alexa Fluor 594 (cat. ab150116; Abcam, Cambridge, UK) for 1 h at room temperature in the dark. Lastly, the cells were incubated with Hoechst Stain (1:200 dilution in PBS). Images were visualized and captured by confocal microscope (BZ-X800, Keyence, Osaka, Japan).

Human liver spheroids were treated with exosomes from RKO cells ovexpressing MAT2A in His-tag or empty vector for 24 h. Next, 16 spheroids per condition were collected from each well (96-well/plate) by 200 µL media containing spheroids into 1.5 mL tube. The tube was placed in a rack for 1 min until all the spheroids settle to the bottom of the tube by gravity. Next, the supernatant was removed and 1 mL of PBS was added to remove residual medium followed by 1000 x g per 30 s. Pellet spheroids into tube were fixed for 30 min at room temperature using 4% paraformaldehyde in PBS at room temperature. Following three washes with PBS, the spheroids were permeabilized with 0.1% Triton-X 100 for 30 min and blocked for 1 h in a blocking buffer containing 1% BSA in PBS. The spheroids were incubated overnight at 4 °C with the anti-MATα1 (cat. PA5-115549; Invitrogen, Waltham, MA) and anti-His tag (cat. 66005-1-Ig; Proteintech, Rosemont, IL) primary antibodies diluted in blocking buffer (1:100). After three more washes with PBS, the spheroids were incubated with rabbit Alexa Fluor 594 (cat. ab150080; Abcam, Cambridge, UK) and mouse Alexa Fluor 488 (cat. ab150117; Abcam, Cambridge, UK) secondary antibodies (1:200) for 1 h at room temperature in the dark. Nuclei were counterstained by incubating the spheroids with 1 µg/mL of Hoechst 33,342 in PBS (cat.62249; Invitrogen, Waltham, MA) for 20 min. Finally, the spheroids were washed three times with PBS and mounted with ProLong Gold antifade reagent (cat. P36930; Invitrogen, Waltham, MA) on BOND 380 slides (ca. IW-T380; IHC World, Ellicott City, MD) for imaging on a confocal microscope (Leica Stellaris 8-STED; Leica, Wetzlar, Germany).

### Human plasma samples, CRLM microarray and immunohistochemistry

Human plasma samples were obtained under approved institutional protocols from Norris Comprehensive Cancer Center at Keck School of Medicine USC. A total of 15 plasma samples were analyzed as described above by western blot: 5 control samples from healthy adult donors with no known history of malignancy and 10 samples from patients diagnosed with CRC. All samples were processed and stored at − 80 °C until analysis.

CRLM tissue microarrays containing 32 CRLM and 3 normal human liver specimens were provided by Core B of P01CA233452. The study protocol conformed to the ethical guidelines of the 1975 Declaration of Helsinki as reflected in a priori approval by the Institutional Review Boards of Cedars-Sinai Medical Center. The slides were deparaffinized, hydrated, and stained for MATα1 (cat. H0004143-M01; Abnova, Taipei City, Taiwan) MATα2 (cat. NB110-94158; Novus, St. Charles, MI), and hepatocyte-specific antigen using extended antigen retrieval (antigen unmasking solution, cat. H-3301-250; Vector Laboratories, Burlingame, CA) as we described [19]. Percent cells staining positive and intensity of staining were separately scored for each specimen as described [24].

### Cell proliferation, migration and invasion

Cell proliferation in RKO cells treated with isolated exosomes was measured by incorporation of 5-ethynyl-2´-deoxyuridine (EdU) into DNA using 647 EdU Click Proliferation Kit (cat. 51-9011284AK; BD Biosciences, San Diego, CA). Briefly, RKO cells were plated in 96-well plate at 0.05 × 10^6^ cells/well and treated with 5uL isolated exosomes after cell attachment. At 20 h treatment, 0.1uL EdU (10 μm) was added to the cell medium, and the reaction was stopped at 24 h. Migration and invasion of RKO cells treated with 5uL isolated exosome from RKO cells overexpressing empty vector or MAT2A were measured as we described [19]. Images of cells were captured using Evos XL Core Imaging System (Thermo Fisher Scientific, Waltham, MA). The images were analyzed using ImageJ.

### Separation of EVs from EV-free media containing MATα2-t

RKO cells were plated 2 × 10^6^ cells/10 cm plate. RKO cells were transfected to overexpress MAT2A or empty vector as described above and cell culture media was replaced 4 h afterwards. After 48 h, cell culture media was collected and centrifuged at 2,000 xg for 20 min at room temperature to remove any debris. Exosomes were precipitated from the culture media as described above. Supernatant from exosome isolation was then collected and dialyzed in culture media to remove exosome precipitation reagent from the media. Slide-A-Lyzer G3 Dialysis Cassettes (cat. A52982; ThermoFisher, Waltham, MA) were soaked in the dialysis fluid to rehydrate the membranes for 2 min. The cassette was then removed from the dialysis fluid and any excess fluid inside the cassette was emptied. 12mL of supernatant from exosome isolation was then added to the cassette and added to the beaker and underwent dialysis at 4˚C while on a magnetic stirrer. After 8 h, the dialysis fluid was replaced, and the samples again underwent dialysis overnight. After dialysis, samples were recovered from the cassettes and concentrated using Amicon Ultra 15 Centrifugal Filters (cat. UFC901008; Merck Millipore, Burlington, MA). Samples were concentrated via centrifugation at 4000 xg for 40 min at 4˚C. After concentration, samples (EV-free media containing MATα2-t) were used for western blot analysis and treatment of RKO cells.

### Treatment with EV-free media containing MATα2-t

RKO cells were plated 0.5 × 10^6^/well in 6-well plates. After 24 h, culture media was replaced with 2mL of EV-free media containing MATα2-t for another 24 h and cell lysates were used for receptor tyrosine kinase array analysis as described below.

### Receptor tyrosine kinase array analysis

Human Receptor Tyrosine Kinase Array Kit was purchased from RayBiotech (cat. AAH-PRTK-1–2; Ray Biotech Life Inc, Peachtree Corners, GA). The kinase array was performed per manufacturer’s protocol. Briefly, 500 µg of cell lysate protein was incubated with the pre‑blocked membrane overnight at 4 °C. After washing, membranes were incubated with a cocktail of biotin-conjugated anti‑phosphotyrosine antibodies, followed by incubation with horseradish peroxidase (HRP)–conjugated streptavidin. Protein phosphorylation signals were detected using enhanced chemiluminescence (ECL) and quantified using the Azure 400 Imaging System (Azure Biosystems, Dublin, CA). Positive and negative controls printed on the array served as internal normalization references.

### Human PBMC isolation and culture

Human peripheral blood mononuclear cells (PBMCs) were isolated from 2 mL whole blood of healthy donor using BD Vacutainer^®^ CPT™ Cell Preparation Tube. Briefly, tube was centrifuged at 1,800 × g for 30 min at room temperature (acceleration 7/deceleration 0). Following centrifugation, the upper plasma layer was carefully removed by aspiration and the resulting white, cloudy PBMC layer was transferred to a fresh 15 mL conical tube. Thereafter, cells were washed by adding 12 mL of pre-warmed (37 °C) PBS and gentlely inverted five times. Next, tube was centrifuged again at 400 x g for 10 min (acceleration 7/deceleration 4) to pellet the cells. The supernatant was discarded, while the cell pellet was resuspended in 5 mL of complete RPMI 1640 culture medium containing 10% FBS, 1% penicillin-streptomycin antibiotics and 1 µg/mL of the T-cell mitogen PHA-L (cat. 00–4977−93; ThermoFisher, Waltham, MA). The resuspended PBMCs were then plated for culture (0.3 × 10^6^/6-well/plate) for 24 h.

### Treatment with neutralizing anti-MATα2 antibody

RKO, HT29and PBMCs were treated with an anti-MATα2 neutralizing antibody from Abcam (cat. ab186129; Cambridge, UK) for 48 h. After the required incubation period, cells were processed for extraction of RNA, protein and TUNEL analyses when indicated.

#### CRISPR-Cas9 gene editing of MAT2A

CRISPR-Cas9 gene editing at the *MAT2A* locus was performed to mutate the canonical (PD) and non-canonical (GXGD) cleaved motifs via homology-directed repair (HDR). Two edit-R synthetic guide RNA sequences (gRNA) were designed to target *MAT2A* PD (chr2:85539343–85539365→ 5’- TTTCACCTCAGAGTCGGTCG-3’) and GXGD (chr2:85539356–85539378→5’- TCGGTCGGGGAAGGCCACCC-3’) motifs, recognized by SpCas9 and synthesized by Horizon (Cambridge, UK). Additionally, a scramble gRNA was also designed and purchased from Horizon. The ssDNA donors were designed using the Edit-R HDR Donor Designer tool to introduce the mutated cleaved motifs (canonical: P30→L30, sequence: 5’- dG.*.dG.*.dC.dA.dC.dA.dT.dT.dC.dC. dT.dT.dT.dT.dC.dA.dC.dC.dT.dC.dA.dG.dA.dG.dT.dC.dG.dG.dT.dC.dG.dG.dG.dG.dA.dA.dG.dG.dC.dC.dA.dC.d.C.dT.dT.dG.dG.dT.dG.dA.dG.dG.dG.dG.dA.dC.dG.dG.dC.dC.dT.dG.dA.dA.dG.dC.dG.dA.dA.dG.dC.dG.dT.*.dG.*.dG-3’; non-canonical: G131→L131 and G133→L133, sequence: 5’- dG.*.dG.*.dC.dA.dC.dA.dT.dT.dC.dC.dT.dT.dT.dT.dC.dA.dC.dC.dT.dC.dA.dG.dA.dG.dT.dC.dG.dG.dT.dC.dC.dT.dG.dG.dA.dA.dC.dT.dG.dC.dA.dC.dC.dC.dA.dG.dG.dT.dG.dA.dG.dG.dG.d.G.dA.dC.dG.dG.dC.dC.dT.dG.dA.dA.dG.dC.dG.dA.dA.dG.dC.dG.dT.*.dG.*.dG-3’) at the targeted *MAT2A* locus. Briefly, 0.5 × 10^6^/6-well/plate or 4 × 10^6^/10 cm-dish RKO or HT29 cells were seeded and transfected pre-incubating the Cas9 plasmid (cat. SKUGE100028; Origine, Rockville, MD) with thesynthetic gRNA to form the complex according to the manufacturer’s protocol. Next, custom ssDNA donor was added to the complex, then introduced into cells using DharmaFECT Duo Transfection Reagent (cat. T-2010-02; Horizon, Cambridge, UK) for 48 h. Next, cells were processed to extract proteins, analyze apoptosis, FAK activation and MAT2A interaction with endopeptidases.Culture media was used to analyze the level of secreted MATα2-t.

### Co-immunoprecipitation

Briefly, 700 µg of total proteins were pre-cleaned using normal mouse IgG-AC (cat. sc-2343, Santa Cruz Biotechnology, Dallas, TX) for 30 min at 4 °C under rotation. Extracted proteins were immunoprecipitated with 3 µg of anti-MAT2A conjugated to agarose (cat. sc-398917 AC, Santa Cruz Biotechnology, Dallas, TX) antibody overnight at 4 °C under rotation. After, protein-antibody complexes attached to the agarose beads were washed five times with 1 ml incubation buffer (150 mM NaCl, 1 mM EDTA, 1 mM EGTA (pH 8.0), 50 mM Tris Cl (pH 7.5), 1% (v/v) Nonidet P-40 (10%, v/v) and 25 mM NaF). Subsequently, 10% SDS-PAGE was used to separate the immunoprecipitated proteins for western blotting, and the blot was probed with anti-DDK (cat. 8146 S; Cell Signaling, Danvers, MA) or anti-PEP (cat. 11536-1-AP; Proteintech, Rosemont, IL) or anti-AEP (cat. 67017-1-Ig; Proteintech, Rosemont, IL) antibodies. VeriBlot (cat. ab131366, Abcam, Cambridge, UK) was used to reduce background noise. Normal IgG (cat. sc2025; Santa Cruz Biotechnology, Dallas, TX) was used as a negative control.

### Measurements of apoptosis

Apoptotic nuclei were detected in HCoEpC, RKO and HT29 cellsby the terminal deoxynucleotidyl transferase-mediated dUTP nick-end labeling (TUNEL) staining using in situ apoptosis detection kit (cat. ab206386; Abcam, Cambridge, UK). Next, the sample was visualized by microscopy (EVOS XL Core, Fisher Scientific, Waltham, MA). Additionally, western blot analyzis was performed to determine the levels of pro-caspase and activated caspase 3 (cat. SE-56053; Santa Cruz Biotechnology, Dallas, TX) to confirm the presence of apoptosis in RKO and HT29 cells.

### Statistical analysis

Data are expressed as mean ± standard error. Data were analyzed using two-tailed unpaired Student’s t-test for comparing two groups and analysis of variance followed by Fisher’s test for multiple comparisons. The ratios of genes or proteins expression levels to housekeeping genes or proteins were calculated. Student’s t-test was used for Pearson correlation. Significant difference was defined by *p* < 0.05.

## Results

### Human CRCs secrete MATα2 via EVs

**Fig. 1:**
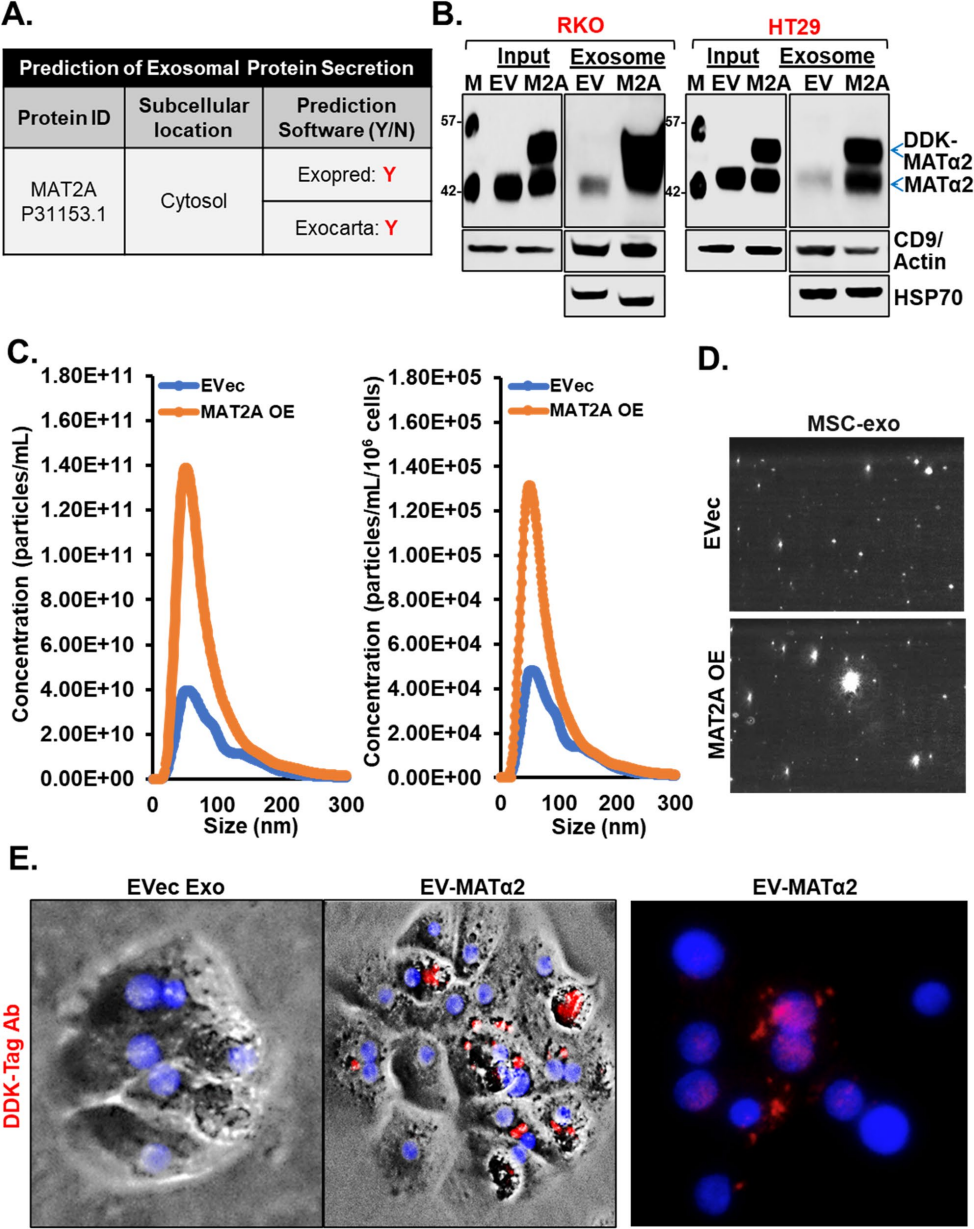
MATα2 is secreted by CRC cells in extracellular vesicles.**A** Exopred and Exocarta software predicts MATα2 is secreted via exosomes (**B**) CRC cells transfected to overexpress DDK-MAT2A (M2A) secreted more MATα2 when compared to empty vector (EV). **C** NanoSight analysis of extracellular vesicles isolated from culture media of CRC cells transfected to overexpress MAT2A (MAT2A OE) and empty vector (EVec) with peak corresponding to size range of exosomes. **D** Images from NanoSight analysis of extracellular vesicles. **E** Immunofluorescence microscopy of human hepatocytes treated with exosomes isolated from culture media from CRC transfected to overexpress DDK-MAT2A and empty vector (EVec) shows internalization of EV-MATα2 with localizing to the nuclues via DAPI staining

We reported MAT1A expression is downregulated in human hepatocytes when cultured with CRC cells [19], suggesting CRC-derived factors may alter MAT1A levels. MAT1A and MAT2A exhibit opposite expression patterns in hepatocytes [25] suggesting a possible reciprocal regulatory mechanism. Based on Exopred and Exocarta software, MATα2 is predicted to be secreted via exosomes (Fig. 1A). To confirm the prediction, RKO and HT29 cells were transfected to overexpress MAT2A in DDK-tag (C-terminal) and EVs were extracted and western blotted, which revealed these CRC cells secrete MATα2 in EVs, which correlated with intracellular level (Fig. 1B). Interestingly, Nanosight analysis of the EVs from RKO cells show more EVs were secreted when cells overexpress MAT2A and the size distribution suggest most are within exosomes (Fig. 1C-D). Although most of EV-MATα2 are within exosomes, it is not 100% so EV-MATα2 will denote all MATα2 within EVs.

### Effect of EV-MATα2 on hepatocytes’ MAT expression

**Fig. 2:**
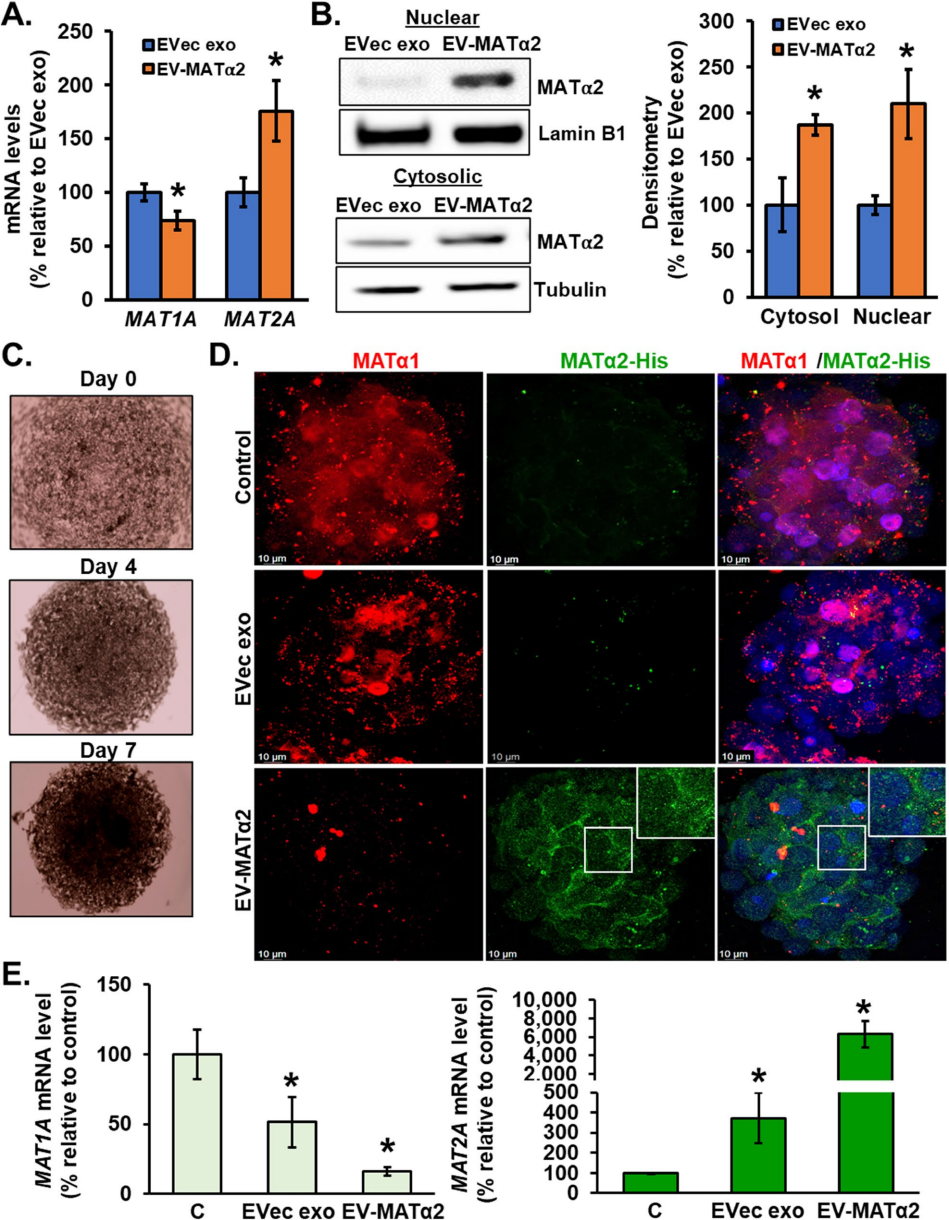
EV-MATα2 is internalized by hepatocytes and alters MAT1A and MAT2A expression**A** mRNA levels of *MAT1A* and *MAT2A* isolated from human hepatocytes treated with exosomes from CRC cells. **B** Western blot analysis of endogenous nuclear and cytosolic MATα2 in human hepatocytes treated with exosomes from CRC cells. Lamin B1 and tubulin were used as loading controls for nuclear and cytoplasmic fractions, respectively. Mean ± SEM from *n* = 3, *p < 0.05 vs. EVec exo. **C-D** Confocal microscopy of human liver spheroids treated with exosomes from CRC cells transfected with empty vector (EVec) or MAT2A-His vector at day 7 for 24 h showing effect on MATα1 and MATα2-His expression. **E** mRNA levels of *MAT1A* and *MAT2A* isolated from human liver spheroids after the exosome treatment. Mean ± SEM from *n* = 3, *p < 0.02 vs.control and †*p* < 0.05 vs. EVec exo

To examine the effect of EV-MATα2 on hepatocytes, we purified exosomes from media of RKO cells overexpressing MAT2A in red DDK tag or empty vector control and treated human hepatocytes for 24 h. MATα2-DDK was visualized inside hepatocytes and hepatocytes’ nuclei (Fig. 1E). At the mRNA level, hepatocytes treated with EV-MATα2 had a fall in *MAT1A* but an increase in *MAT2A* (Fig. 2A). Furthermore, cell fractionation and western blot analysis illustrated an increase in endogenous cytosolic and nuclear MATα2 after treatment with EV-MATα2 as compared to exosomes fom empty vector control (Fig. 2B). The same changes were also observed in human hepatocytes spheroids treated with EV-MATα2 (His-tag) as compared to exosomes from empty vector control and revealed clear intracellular accumulation of MATα2-His, indicating the internalization of MATα2-His-containing EVs by hepatocytes (Fig. 2C-E).

### MATα2 chromatin immunoprecipitation-sequencing (ChIP-Seq) analysis in CRC

**Fig. 3:**
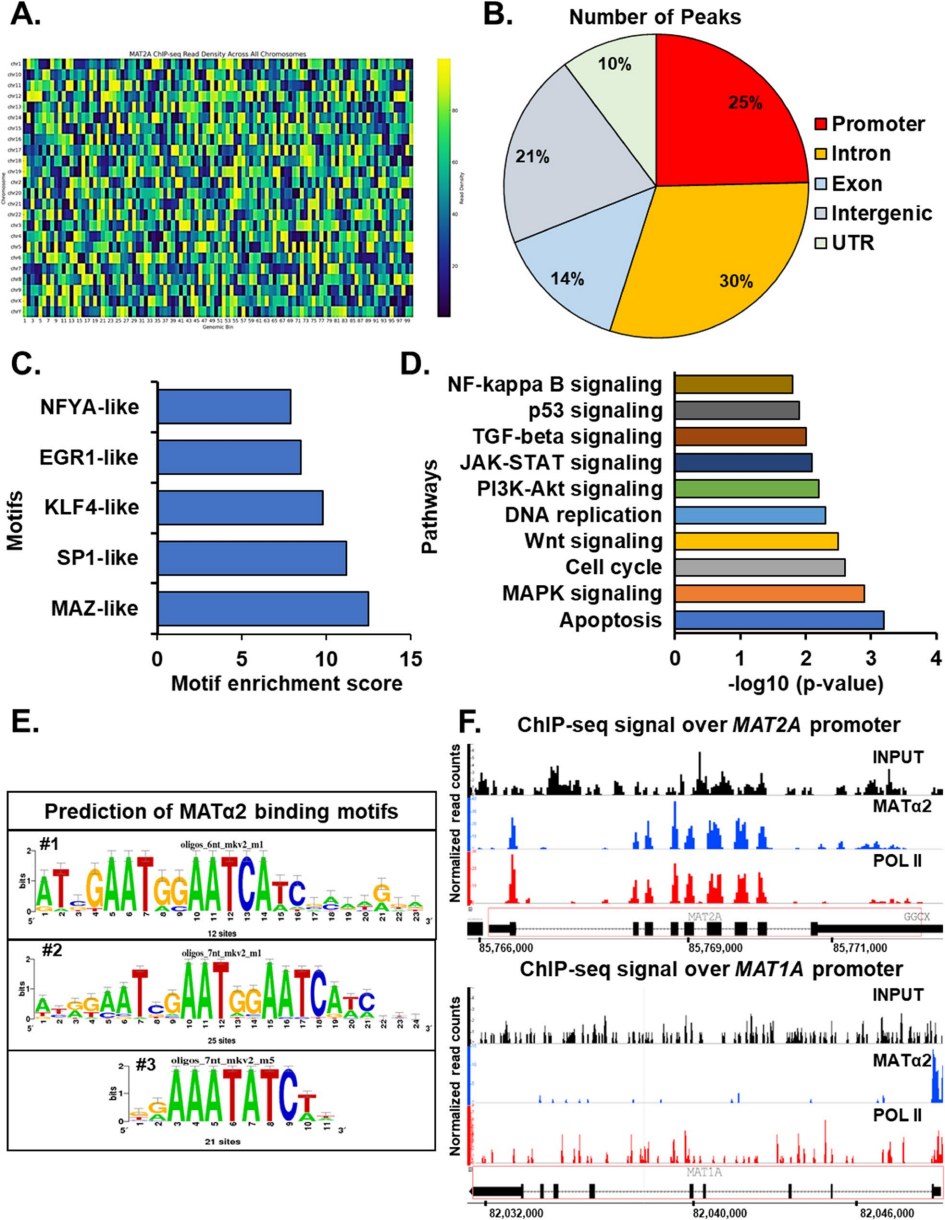
Integrated analysis of MATα2 genomic binding profiles in CRC cells.**A** Heatmap showing the read density distribution of MATα2 ChIP-seq peaks across all human chromosomes. Read intensities are color-coded from low (purple) to high (yellow) density. **B** Genomic annotation of MATα2 binding peaks. **C** Top five DNA-binding motifs enriched within MATα2-bound peaks ranked by motif enrichment score. **D** Pathway enrichment analysis of MATα2-associated genes. **E** Predicted consensus sequences logos representing the top three de novo motifs identified from MATα2 binding sites by Jaspar software. **F** ChIP-seq tracks showing MATα2 (blue), RNA polymerase II (POL II; red) and input control (black) signals across the *MAT1A* (lower) or *MAT2A* (upper) locus. The Y-axis represents normalized read enrichment. The bottom track shows the gene structure with exons (black boxes) and introns (dashed lines)

ChIP-seq analysis identified a total of 2,356 high-confidence MATα2 binding peaks across the genome (Fig. 3A). Data have been uploaded to the BioStudies database (www.ebi.ac.uk/biostudies/studies/S-BSST2200).The majority of binding sites were located in promoter (28%) and intronic regions (34%), with additional peaks mapped to exonic (15%), intergenic (20%), and UTR regions (3%) (Fig. 3B). Further motif enrichment analysis against known transcription factor binding profiles revealed a strong enrichment of NFYA-like, EGR1-like, KLF4-like, SP1-like, and MAZ-like motifs among MAT2A peaks (Fig. 3C), suggesting potential cooperation or competition between MATα2 and these transcriptional regulators. Pathway analysis of genes associated with MATα2 binding peaks revealed significant enrichment in pathways regulating apoptosis, MAPK signaling, cell cycle control, Wnt signaling, and DNA damage response (Fig. 3D). These findings suggest that MATα2 may play a direct role in modulating oncogenic and stress-response transcriptional programs in CRC cells.

To uncover potential DNA sequence preferences of MATα2, we performed de novo motif discovery analysis on the MATα2 ChIP-seq peaks identified in CRC cells that was set with RSAT software revealing enrichment for several motifs [21]. The first motif, identified from 12 enriched sites, is characterized by a core GAA[T/G] sequence followed by a conserved GGAA[T/C] tract, indicative of a strong purine-rich binding preference (Fig. 3E). The second motif, found in 25 enriched sites, similarly displayed a consensus GGAATCA core, suggesting that MATα2 may preferentially bind to tandem repeats of G/A-rich sequences, reinforcing the specificity observed in the first motif (Fig. 3E). The third motif, derived from 21 sites, highlighted an even more conserved AAATATC sequence pattern, which may represent a high-affinity binding site or a variant motif under specific chromatin contexts (Fig. 3E). The high conservation of observed motifs implies that MATα2 binding may play a regulatory role at select genomic loci.

We also found an important MATα2 enrichment at the *MAT2A* promoter region (85,765,291–85,767,291, hg19) (Fig. 3F, upper panel). A similar pattern of binding was discovered at the *MAT1A* promoter (chr10:82,048,414–82,050,414, hg19) (Fig. 3F, lower panel), suggesting a direct association of MATα2 with regulatory elements in the promoter regions of both genes. These regions exhibited strong ChIP-seq signals compared to input and overlapped with defined peak regions. Notably, POL II ChIP-seq analysis revealed overlapping signal peaks with MATα2, demonstrating that MATα2 binds within transcriptionally active chromatin domains. This spatial correlation supports a transcription factor–like role for MATα2, implicating it in the regulation of gene transcription. In addition, these findings suggest a potential autoregulatory loop at the *MAT2A* locus and possible transcriptional repression of *MAT1A* by MATα2 binding.

### EV-MATα2 directly binds and regulates *MAT1A *and *MAT2A *promoters

Using the Integrated Genome Viewer (IGV) and UCSC Genome Browser tracks, we found a dense clusters of MATα2 binding at promoter regions of key regulatory genes, including *MAT1A* and *MAT2A* itself, supporting a potential autoregulatory mechanism (Fig. 3F). Broader genome-wide visualization indicated that MATα2 binding preferentially occurred near transcriptionally active chromatin domains, corroborating our peak annotation analysis.

**Fig. 4:**
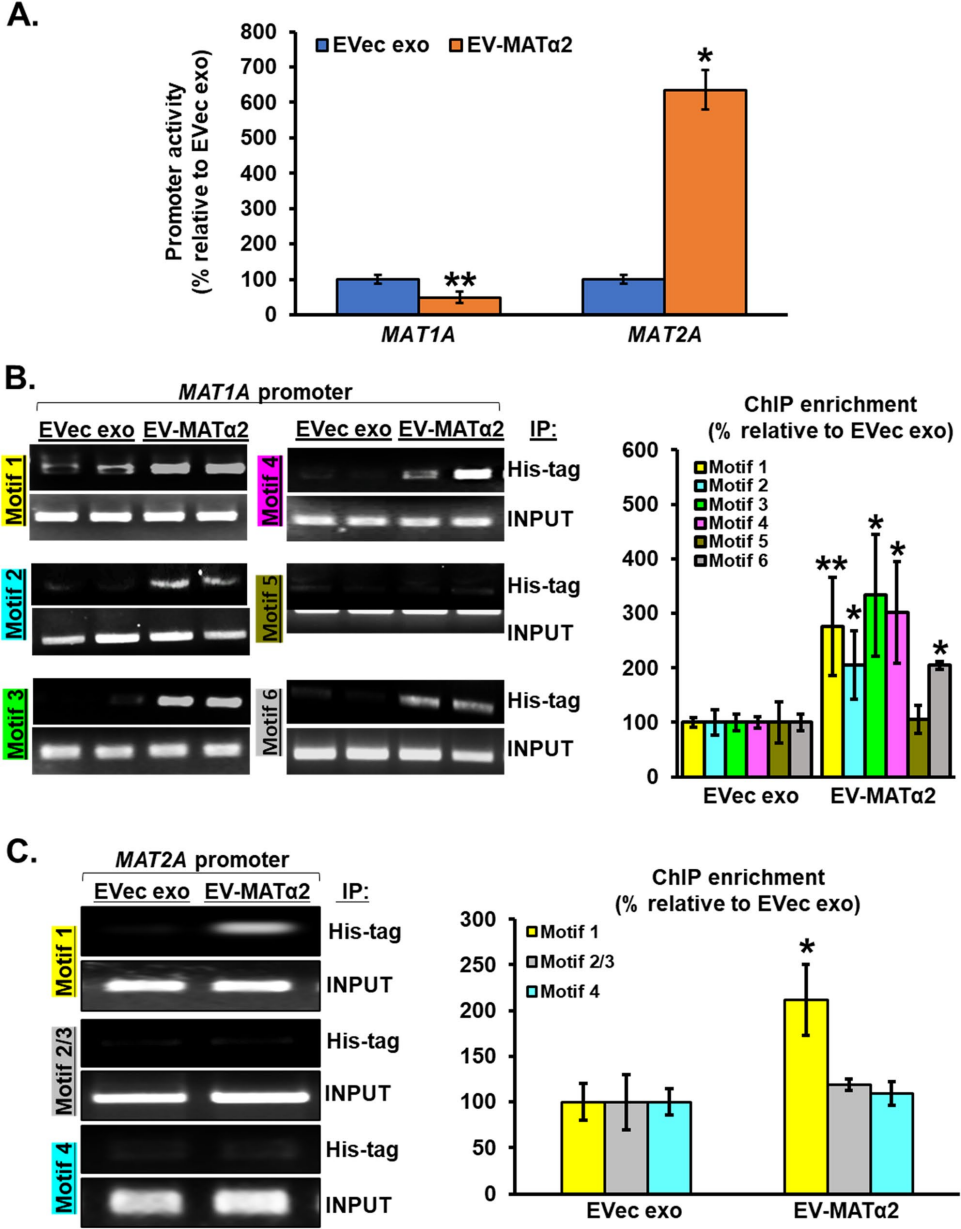
EV-MATα2 acts as a transcription factor to alter *MAT1A *and *MAT2A *expression.** A** Promoter activities in human hepatocytes transfected with human *MAT1A* or *MAT2A* promoter constructs and then treated with exosomes from RKO cells expressing empty vector (EVec exo) or MAT2A (EV-MATα2) as described in Methods. **B** ChIP analysis of the human *MAT1A* and (**C**) human *MAT2A* promoters showing binding of MATα2-His to different regions of the promoters. Mean ± SEM from *n* = 8, *p < 0.05 and **p < 0.01 vs. EVec exo for *MAT1A* promoter; *n* = 7, *p < 0.04 vs. EVec exo for *MAT2A* promoter

To investigate the functional consequences of EV-MATα2 on hepatocyte gene regulation, we assessed *MAT1A* and *MAT2A* promoter activity in hepatocytes treated for 24 h with exosomes isolated from either empty vector or MAT2A-overexpressing (OE) RKO cells. A significant 60% decrease in *MAT1A* promoter activity was observed following treatment exosomes from MAT2A-OE cells compared to empty vector controls (Fig. 4A). Conversely, *MAT2A* promoter activity was increased by approximately 500% (Fig. 4A). RSAT software was used to predict the possible binding motifs of MATα2, which were then identified in the promoter regions of *MAT1A* and *MAT2A*. After the binding motifs were identified (Supplemental Fig. 1A-B), primers were designed to flank each binding motif (Supplemental Table 2).

Next, human hepatocytes were treated with exosomes from RKO cells expressing empty vector or DDK-MAT2A-His Tag vector. Then, ChIP was done using His-Tag antibody and the eluted ChIP DNA was used for PCR with the primers designed for *MAT1A*/*MAT2A* promoter regions. EV-MATα2-His was binding to the *MAT1A* promoter specifically in the regions of motifs 1, 2, 3, 4, and 6 and to *MAT2A* promoter in the region of motif 1 (Fig. 4B-C). No binding occurred with primers for *MAT1A* promoter motif 5 or for MAT2A promoter motif 2/3 and 4. Thus, the internalized EV-MATα2 can act as a transcription factor to suppress *MAT1A* while activating *MAT2A* at the promoter level.

### EV from MAT2A OE CRC cells can further induce oncogenic activity

**Fig. 5:**
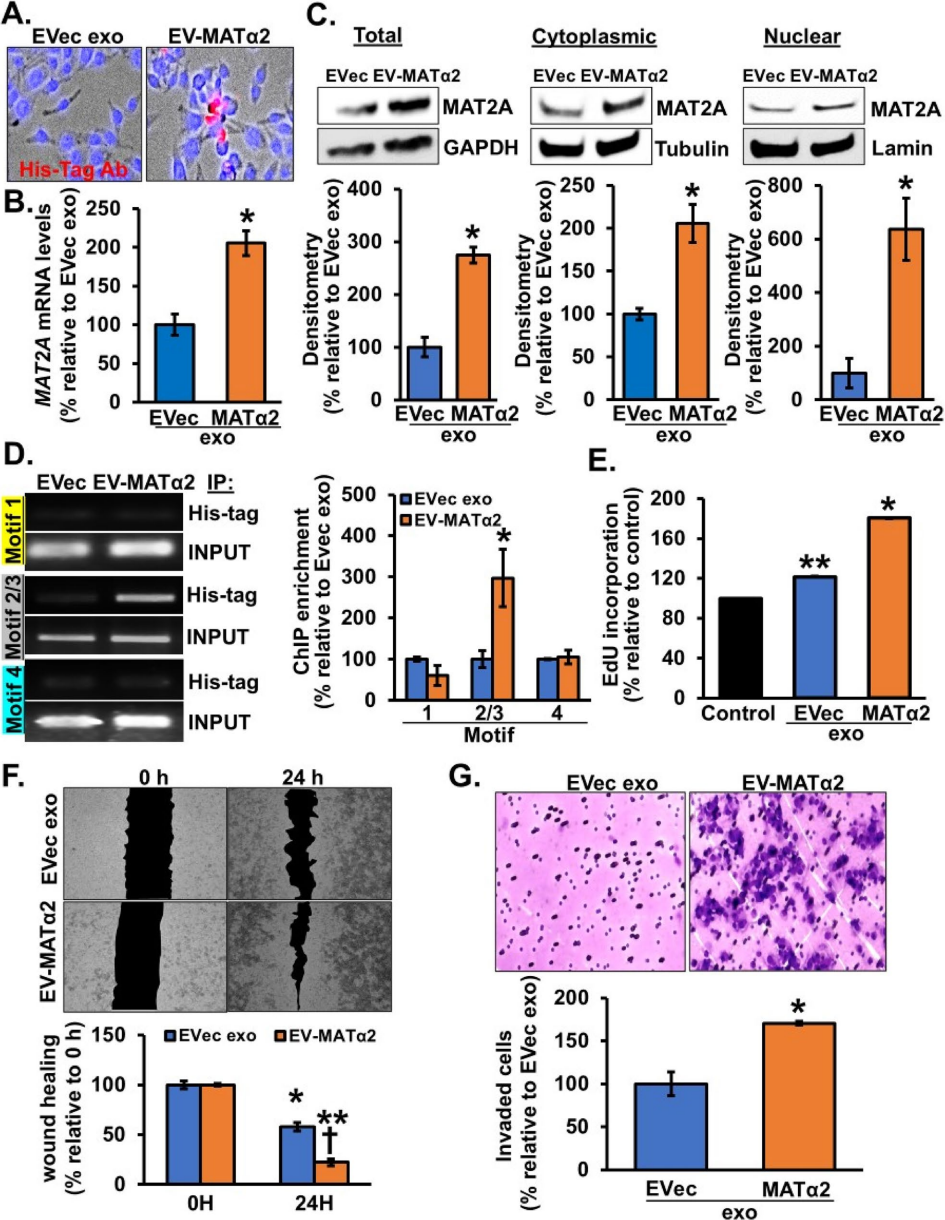
EV-MATα2 induces MAT2A expression and oncogenic activity in RKO cells. **A** RKO cells were treated with exosomes from RKO cells expressing empty vector (EVec exo) or MAT2A-His-Tag vector (EV-MATα2) as described in Methods and cell entry was visualized under fluorescent microscopy using His-tag antibody. **B** Real-time PCR shows the effect at the *MAT2A* mRNA level. Mean ± SEM from *n* = 3, *p < 0.02 vs. EVec exo. **C** Western blotting was done in total cell lysate, cytoplasmic and nuclear fractions showing increased MATα2 levels. Densitometry were measured by ImageJ. Mean ± SEM from *n* = 3, *p < 0.002 for total lysate, *p < 0.02 for cytoplasmic, *p < 0.03 for nuclear fractions vs. EVec exo. **D** ChIP analysis of the human *MAT2A* promoter showing binding of MATα2-His to different predicted motifs. Mean ± SEM from *n* = 3, *p < 0.05 vs. EVec exo. Effects of the same treatments on EdU (**E**) (*n* = 3, *p < 0.05 and **p < 0.01 vs. control), migration (**F**) (*n* = 3, *p < 0.004 and **p < 0.0001 vs. 0 h EVec exo, †*p* < 0.003 vs. 24 h EV-MATα2), and invasion (**G**) (*n* = 3, *p < 0.008 vs. EVec exo)

To see if EV-MATα2 can further augment MAT2A expression in an autocrine/paracrine fashion, RKO or HT29 cells were treated with exosomes from RKO or HT29 cells expressing empty vector or DDK-MAT2A-His Tag vector. ChIP was done using His-Tag antibody and eluted ChIP DNA was used for PCR with the same primers. EV-MATα2 was internalized by RKO cells (Fig. 5A), which resulted in higher MAT2A expression at the mRNA and protein levels (Fig. 5B-C). EV-MATα2 was found to bind *MAT2A* promoter specifically in the region of motif 2/3 (Fig. 5D) (Supplemental Fig. 2). These results showed that EV-MATα2 physically interacts with *MAT2A* promoter in colon cancer cells to regulate its promoter activity.

To further explore the functional significance of EV-MATα2, we measured colon cancer cell proliferation, migration, and invasion after treatment with exosomes from RKO cells overexpressing MAT2A. There was a 20% increase in cell proliferation in cells treated with exosomes from empty vector RKO compared to control and a 80% increase in cells treated with exosomes from MAT2A OE RKO cells (Fig. 5E). Similarly, exosomes from MAT2A OE RKO cells increased migration and invasion significantly as compared to exosomes from empty vector expressing RKO cells (Fig. 5F).

### CRLM alter MAT expression in adjacent hepatocytes

**Fig. 6:**
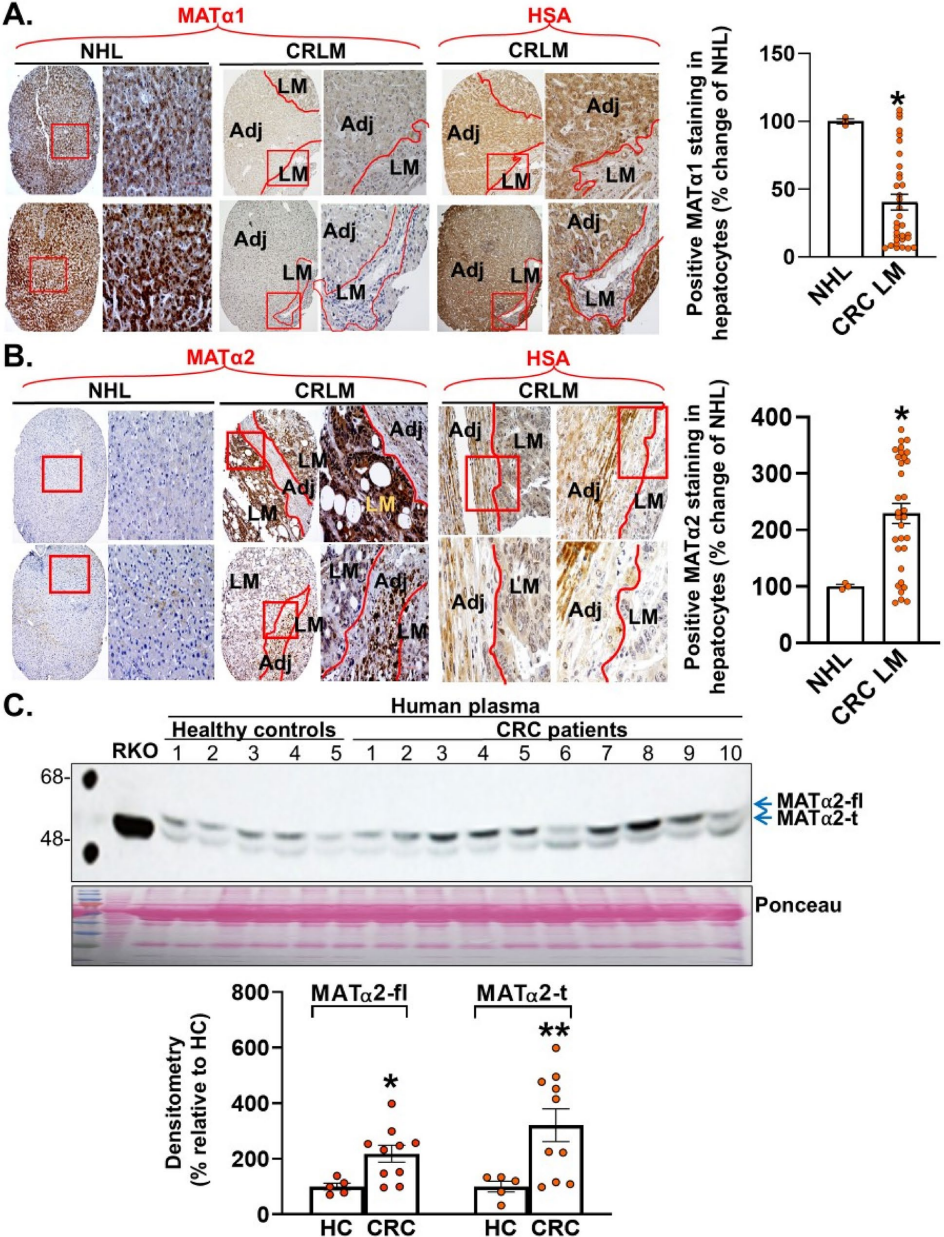
MAT expression in adjacent hepatocytes is altered in human CRLM and patients with CRC secrete two forms of MATα2.** A**, **B** Human tissue microarray including normal human liver (NHL, *n* = 3) and CRLM (*n* = 32) were examined using IHC (x100) for MATα1, MATα2, and hepatocyte specific antigen (HSA). Boxed areas are magnified, MATα1 and MATα2 staining in hepatocytes was analyzed by Image J and summarized in the graphs. Mean ± SEM, *p < 0.005 for MATα1, *p < 0.04 for MATα2 vs. NHL. **C** Human plasma from 5 healthy controls (HC) and 10 CRC patients were western blotted for MATα2 to detect full-length MATα2 (MATα2-fl) and truncated MATα2 (MATα2-t), with RKO lysate as input control. Mean ± SEM. *p < 0.05 vs. HC, **p < 0.001 vs. HC

To see if the changes we observed in vitro is true in human CRLM, tissue microarrays from CRLM (*n* = 32) and normal human liver (*n* = 3) were examined for MATα1 and MATα2 expression using immunohistochemistry (IHC) (Fig. 6A-B). There was a 60% decrease in MATα1 staining in hepatocytes adjacent to CRLM compared to normal healthy liver as well as a 150% increase in MATα2 staining in hepatocytes adjacent to CRLM compared to normal healthy liver. We used hepatocyte-specific antigen to ensure cells examined were hepatocytes. This confirms our in vitro findings and that in human CRLM, presence of CRC in the liver resulted in lower MATα1 but higher MATα2 expression in hepatocytes of non-tumor regions.

### Cancer cells also freely secrete a novel truncated form of MATα2 (MATα2-t)

**Fig. 7:**
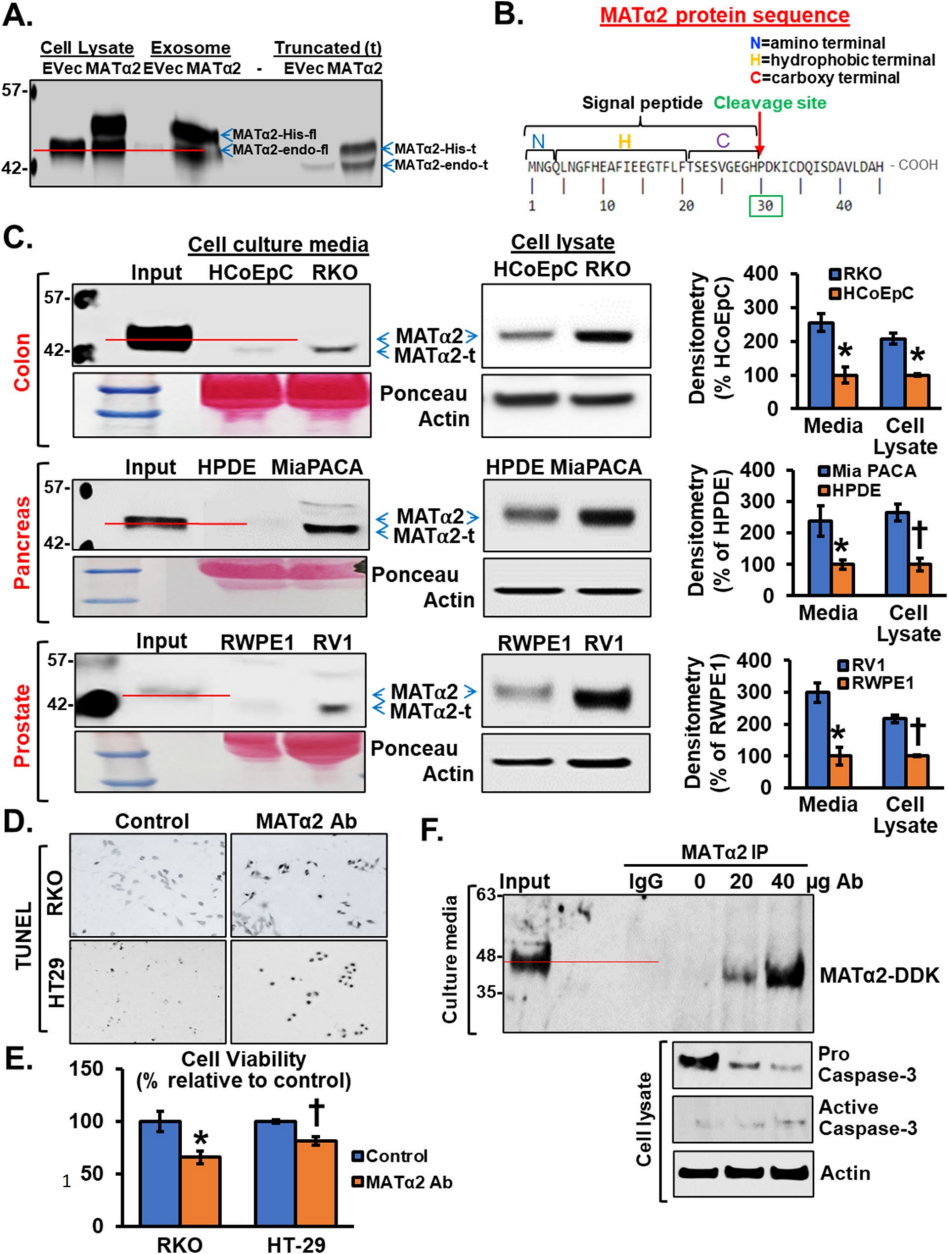
Cancer cells secrete more truncated MATα2, which is required for survival.** A** Medium from RKO cells overexpressing MAT2A-His or empty vector (EVec) was separated into exosomes and EV-free media that only has truncated MATα2 (MATα2-t). Note MATα2-His has higher MW than full length endogenous MATα2, which has the same MW as MATα2-t-His. Endogenous MATα2-t has the lowest MW. **B** MATα2 protein sequence and predicted cleavage site based on PrediSI is at proline 30. **C** RKO (CRC), MiaPACA (pancreatic adenocarcinoma) and RV1 (prostate adenocarcinoma) cells secrete more MATα2-t as compared to the respective non-malignant cells (HCoEpC, HPDE, RWPE1). Mean ± SEM from *n* = 3, *p < 0.01 vs. HCoEpC cells; *p < 0.03 and †*p* < 0.01 vs. HPDE cells; *p < 0.03 and †*p* < 0.001 vs. RWPE1 cells. **D** RKO and HT29 cells treated with anti-MATα2 (20 µg/ml) for 48 h and TUNEL staining shows CRC cells underwent apoptosis. **E** MTT assay in RKO and HT29 cells treated with anti-MATα2 shows a fall in viability. Mean ± SEM from *n* = 3, *p < 0.03 and †*p* < 0.04 vs. control. **F** RKO cells were transfected with MATα2-DDK for 48 h and increasing amount of anti- MATα2 Ab was added, followed by pull-down of MATα2 Ab using beads, then western blotted the pull-down with anti-DDK Ab. The MATα2 antibody was able to bring down freely secreted MATα2 in a dose-dependent manner. This correlated with a dose-dependent increase in active caspase 3

We also examined human plasma samples from patients with CRC as compared to normal controls and noticed that in addition to the more abundant full-length MATα2 in plasma samples of CRC patients, there is a truncated forms of MATα2 (MATα2-t) that is also at a much higher levels as compared to normal healthy controls (Fig. 6C). To examine this further, we overexpressed RKO cells with MAT2A in His tag and empty vector control, separated EVs from EV-free media. We found MATα2 in the exosomes is full-length, similar to MATα2 in cell lysates (addition of His tag increased MW). MATα2-t is present only in the EV-free media (Fig. 7A). From Predisi prediction software, MATα2 is predicted to be cleaved at amino acid 30 (Fig. 7B). To demonstrate that the secreted truncated MATα2 is an N-terminal cleaved product rather than an artifact of tag positioning or aberrant translation initiation, we overexpressed MAT2A in DDK(N-terminal)-His(C-terminal)-tag in RKO cells. We found the intracellular full-length of MATα2 retained both tags whereas the truncated MATα2 detected in culture media lacked the DDK signal but preserved the His-tag, confirming loss of the N-terminal portion (Supplemental Fig. 3). When compared to non-malignant epithelial cells from the colon, pancreas, and prostate, cancerous cells secrete more of the MATα2-t and in contrast to plasma samples, secrete mainly the MATα2-t form (Fig. 7C, Supplemental Fig. 4).

### Secreted MATα2-t is essential for CRC cell survival

**Fig. 8:**
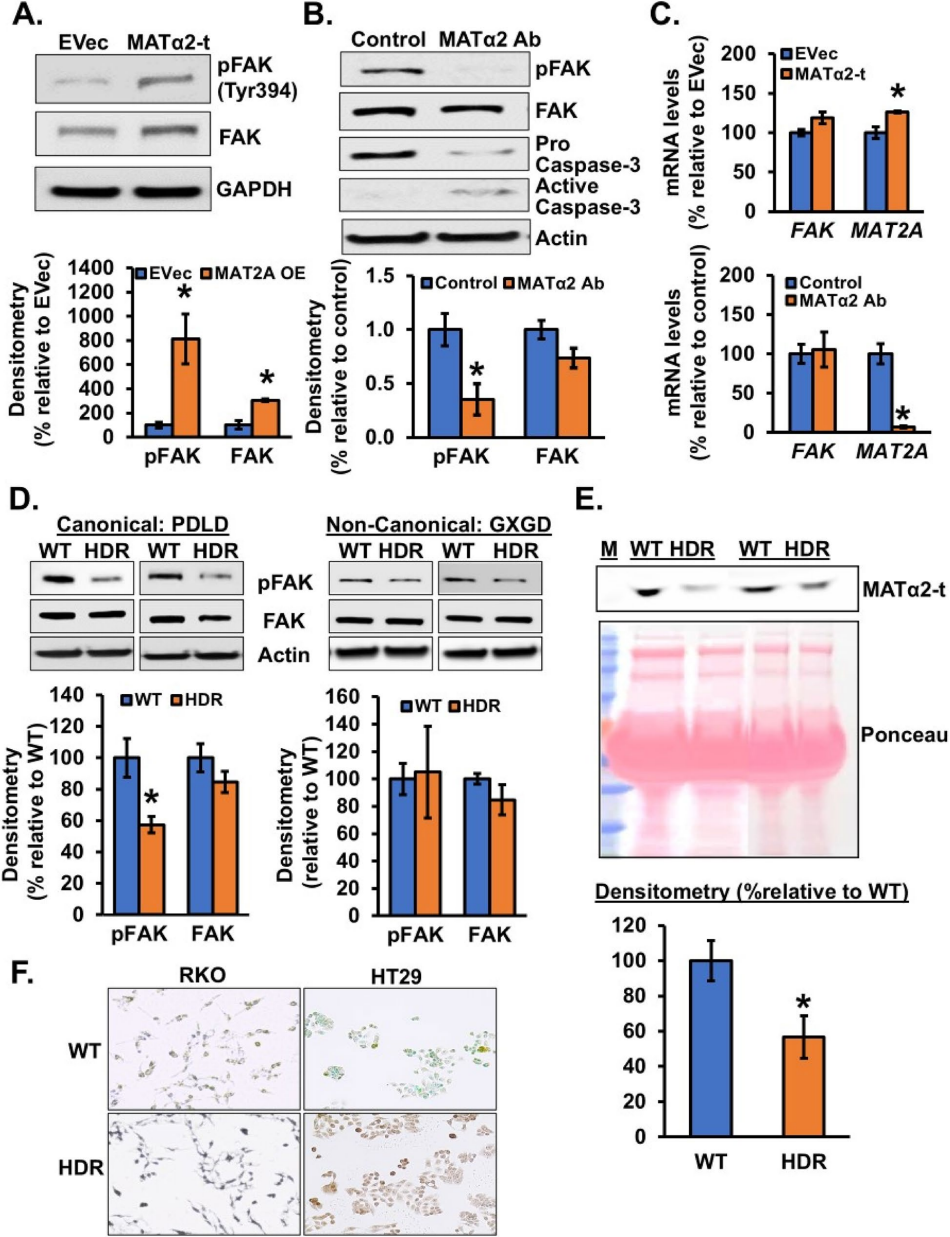
Secreted MATα2-t activates FAK and is required to maintain *MAT2A *expression. **A** RKO cells were treated with EV-free media containing MATα2-t as described in Methods and western blotted for pFAK and total FAK. Mean ± SEM from *n* = 3, *p < 0.04 vs. EVec (**B**) RKO cells were treated with anti-MATα2 Ab for 48 h and western blotted for pFAK, total FAK, pro-caspase 3 and active caspase 3. Mean ± SEM from *n* = 3, *p < 0.03 vs. control. **C** *FAK* and *MAT2A* mRNA levels in RKO cells from the above treatments were measured by real-time PCR. Mean ± SEM from *n* = 3, *p < 0.002 vs. EVec; *p < 0.002 vs. control. **D** RKO cells were CRISPR/Cas9 gene edited (HDR) to mutate proline to leucine at position 30 (canonical motif: PDLD) and at positions 131 and 133 glycine to leucine (non-canonical motif: GXGD); western blotted for pFAK and FAK. Mean ± SEM from *n* = 5–6, *p < 0.01 vs. wild-type (WT) for PDLD. **E** Immunoblotting of secreated MATα2 (MATα2-t) in culture media from RKO cells gene edited PDLD motif. Mean ± SEM from *n* = 3, *p < 0.04 vs. WT. **F** PDLD gene edited RKO and HT29 cells exhibited increased apoptosis on TUNEL staining (blue for RKO and brown for HT29 depending to pH culture media)

To examine the function of the secreted MATα2-t we treated RKO and HT29 cells with neutralizing antibody to MATα2 for 24 h. Surprisingly, we found an increase in apoptotic cell death (Fig. 7D-F). Importantly, this did not occur in human PBMCs or non-malignant colon epithelial cells (Supplemental Fig. 5A). To gain insight on how MATα2-t regulates CRC survival, we hypothesized that the secreted MATα2-t might act like a ligand to activate membrane receptors. Using a kinase array, we found treatment of RKO cells with EV-free media containing MATα2-t resulted in activation of focal adhesion kinase (FAK) (Table 1), which is known to be overexpressed in many cancers and promote cancer cell survival [26]. Consistently, treatment of RKO cells with EV-free media containing MATα2-t activated FAK, whereas neutralizing antibody to MATα2 in both RKO and HT29 cells inhibited FAK activity and activated apoptosis (Fig. 8A-B, Supplemental Fig. 5B). Treatment with MATα2-t also resulted in a small rise in *MAT2A* mRNA level, whereas neutralizing antibody to MATα2 had a dramatic inhibitory effect on *MAT2A* mRNA levels. *FAK* mRNA levels were not significantly affected (Fig. 8C). These results suggest MATα2-t is required to maintain its own expression in RKO cells, possibly through FAK.

**Table 1.**
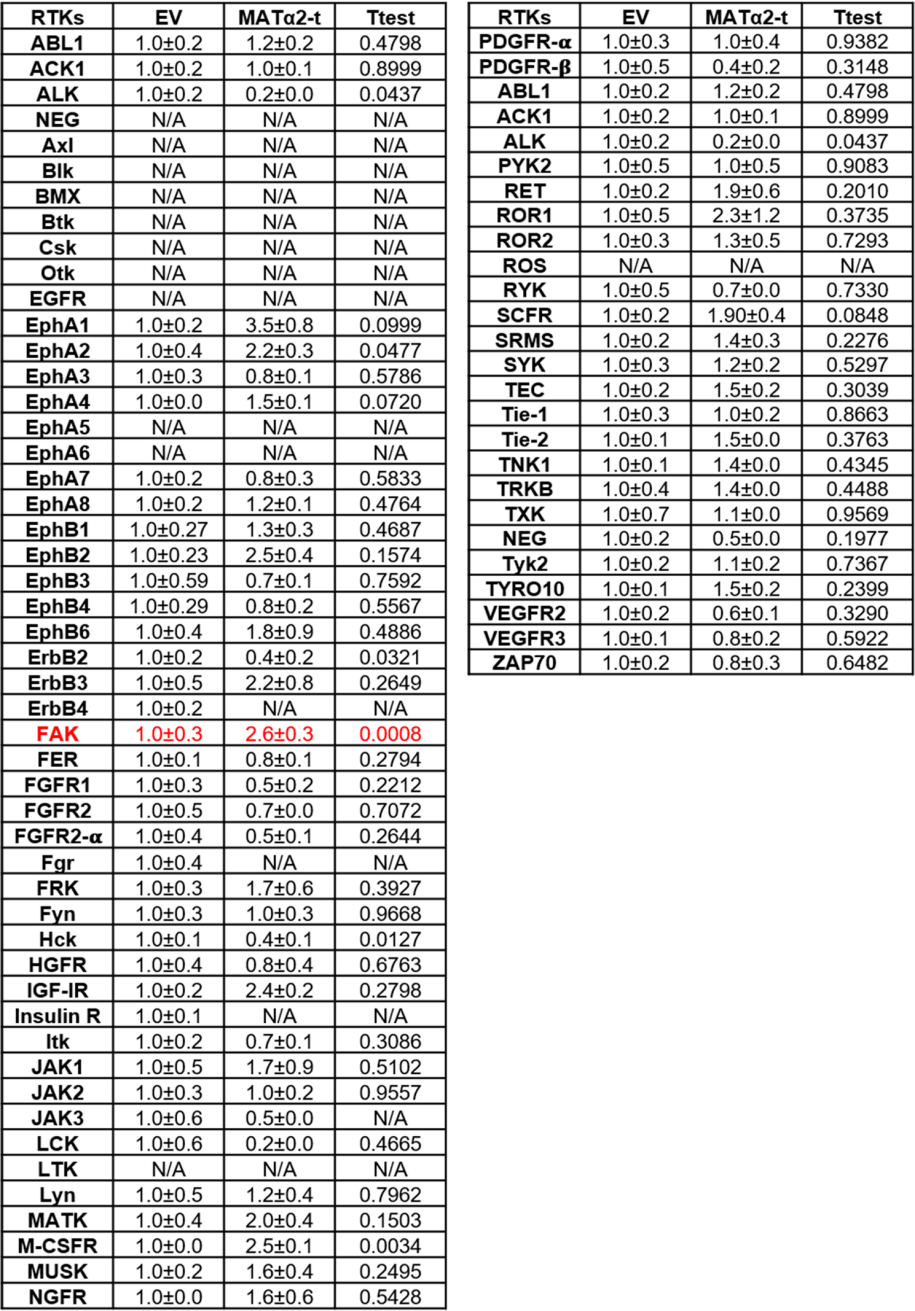
Phosphorylation of RTKs in RKO cells treated with MATα2-t

Two peptidases are predicted to cleave MATα2 at position 30 - asparaginyl endopeptidase (AEP) and prolyl endopeptidase (PEP). Both are overexpressed in many cancers and are being targeted in cancer therapy [27, 28]. MATα2 interacts with both peptidases and overexpressing MAT2A increased their interaction in both RKO and HT29 cells (Supplemental Fig. 6A). To definitively examine the importance of this cleavage, MATα2 was gene-edited by CRISPR/Cas9 of the canonical cleavage motif (PDLD) at position 30 from proline (P30) to leucine (L30). Western blot analysis of wild-type (WT) and homozygous donor-repaired (HDR) cells proved that P30L mutation impaired FAK activation showing decresed pFAK level compared to WT, without affecting total FAK level (Fig. 8D, left panel, Supplemental Fig. 5B). In contrast, mutating the non-canonical cleavage motif (GXGD) at positions 131 and 133 glycine (G131 and G133) to leucine (L131 and L133) showed no change in FAK signaling (Fig. 8D, right panel). This finding indicates a sequence-specific dependence of MATα2 cleavage for FAK activation. To verify whether P30L mutation affects MATα2 secretion, we performed western blot analysis on culture media from gene-edited RKO and HT29 cells reveling a substantial reduction in secreted MATα2-t in HDR compared to WT cells (Fig. 8E, Supplemental Fig. 5C). To evaluate if MATα2 secretion influences CRC cells survival, we performed TUNEL staining in WT and HDR cells finding a marked increased staining in positive cells (Fig. 8F) that indicates increased apoptosis upon disruption of MATα2 cleavage motif. Finally, we examined whether disrupting the MATα2 cleavage motif affected PEP and AEP binding, we compared WT and HDR-edited RKO cells by co-immunoprecipitation. In HDR cells, MATα2 association with both peptidases was preserved indicating that the mutated cleavage site did not impair binding (Supplemental Fig. 6B).

## Discussion

Liver is the most common site of metastasis for multiple cancers, with CRC topping the list. Presence of liver metastasis lowers overall survival and often leads to treatment resistance [29]. Liver metastasis is a multi-stage complex process that starts with cancer cell intravasation and arrest in the sinusoids, an extravascular phase prior to angiogenesis, angiogenesis phase, and finally the growth phase [30]. Crosstalks between the cancer cell and the liver occur prior to cancer cell seeding and continue during this process. Extensive studies have focused on cancer-related factors that promote liver metastasis [31]. Recently, intrinsic liver factors have been reported to influence CRLM. This includes steatotic liver, which promotes liver metastasis [32] and more recently we reported loss of either MAT1A or prohibitin 1 (PHB1) sensitized the liver to CRLM through a metalloproteinase 7 (MMP-7)-dependent mechanism [19]. The mechanism lies at the ability of MATα1 and PHB1 to repress MMP-7 at the transcriptional level and when MMP-7 induction was blocked, the sensitization to CRLM was lost [19]. Interesting, while normal hepatocytes typically express almost no MMP-7, presence of CRLM induced MMP-7 expression in hepatocytes far away from CRLM. Furthermore, when co-cultured with RKO cells, MAT1A expression fell dramatically while MMP-7 expression was induced [19]. These findings led us to hypothesize that CRCs release factor(s) to lower hepatocytes’ MAT1A expression, allowing for induction of MMP-7 to occur and facilitating metastatic growth. In this study we focused on MATα2 because we have observed that in hepatocytes, MAT1A and MAT2A expression patterns are opposite of each other, suggesting a reciprocal regulation [10]. Furthermore, MATα2 is reported to be present in circulation on the Human Protein Atlas (*MAT2A protein expression summary - The Human Protein Atlas*). In the course of our study, we confirmed that MATα2 is secreted and uncovered two new aspects of MATα2 biology: first, that EV-MATα2 can be internalized and translocate to the nucleus to act as a transcription factor, and second, a previously unreported truncated form of MATα2 (MATα2-t) is preferentially released by cancer cells and is essential for cancer cell survival in part by activating FAK. These findings have important implications with regards to CRLM.

Studies have shown that MAT2A is overexpressed in CRC and is associated with growth and increased survival of CRC [13, 33]; however, no studies have shown a specific pathway for secreted MATα2 to promote CRLM. In agreement with the Human Protein Atlas, we found cancer cells (colon, pancreas, and prostate) secrete MATα2 at a higher amount relative to its intracellular content as compared to non-malignant epithelial cells from the same organs. Furthermore, MATα2 is secreted in two forms, one is within EV, which upon characterization suggest most is present in exosomes; the other is free but truncated at amino acid 30. Both play complementary but important roles to promote CRLM. Importantly, CRC patients have higher plasma levels of the full-length and truncated MATα2 forms, supporting the importance of our findings. One major difference in MATα2 secretion is that, whereas the full-length (presumably within EVs) seem to more abundant in the plasma samples, cancer cells mainly secrete the truncated form. This may be because plasma MATα2 reflects secretion from all cells in the body, whereas cancer cells have higher MATα2 content and increased expression/activity of the peptidases that cleave MATα2.

To study the influence of EV-MATα2, we used an overexpression vector that has a fluorescent tag, which allowed us to track its location. With this approach we verified that exosomal MATα2 can be internalized and bind to promoter regions of *MAT1A* and *MAT2A* to regulate their promoter activities, repressing the former while activating the latter. This explains the reciprocal regulatory pattern often observed. Since hepatic MAT1A is a major defense against CRLM, suppression of its expression will facilitate CRLM. EV-MATα2 also exerts an autocrine effect on CRCs, as it promoted growth, migration and invasion. Importantly, we confirmed the alterations of MAT expression in vivo with CRLM tissue microarrays showing decreased MATα1 and increased MATα2 expression in hepatic parenchyma adjacent to colorectal metastases.

However, EV-MATα2 likely has a much wider influence beyond *MAT1A*/*MAT2A*. This is suggested by MATα2 ChIP-seq results, which revealed a strong enrichment of NFYA-like, EGR1-like, KLF4-like, SP1-like, and MAZ-like motifs among MATα2 peaks, suggesting potential cooperation or competition between MATα2 and these transcriptional regulators. Pathway analysis of genes associated with MATα2 binding peaks revealed significant enrichment in pathways regulating apoptosis, MAPK signaling, cell cycle control, Wnt signaling, and DNA damage response. These findings suggest that MATα2 may play a direct role in modulating oncogenic and stress-response transcriptional programs in CRC cells. Further studies will be required to investigate these.

To study the function of MATα2-t we first treated cells with neutralizing antibody to MATα2, which would only affect the freely secreted form and not the one within EVs. We were surprised to find that this resulted in apoptosis of CRCs, but not non-malignant colon epithelial cells and human PBMCs. This may be due to the higher expression of MATα2 and increased release of the truncated form. Intrigued by this finding we explored possible mechanisms first using a kinase array, which revealed activation of FAK when treated with EV-free media containing MATα2-t. FAK is well known to promote cancer cell survival and is a poor prognostic marker in CRC [34]. We further confirmed that neutralizing antibody to MATα2 abolished FAK activity while activating apoptosis. The intermediate steps between the released MATα2-t and activation of FAK remains unknown and is a topic of future investigation. Neutralizing antibody to MATα2 also dramatically lowered *MAT2A* mRNA level. Although there is no literature showing FAK regulates MAT2A expression, the signaling pathways regulated by FAK such as PI3K/AKT and MAPK [26] are known to regulate MAT2A positively [35]. Future studies are needed to confirm the causal relationship between FAK and MAT2A expression. Taken together, cancer cells release a previously unrecognized MATα2-t that exerts an autocrine effect to support its own survival.

Lastly, the question arose as to which peptidase(s) are responsible for cleaving MATα2. Based on predication software, AEP and PEP are predicted to cleave MATα2 at amino acid 30. Both are known to be overexpressed/activated in multiple cancers [28, 36]. Consistently, we found MATα2 interacts with both and there is increased interaction when MAT2A was overexpressed. More importantly, we found that when the site was gene edited, there was a reduction in the release of truncated MATα2, FAK activation, which coincided with an increase in apoptosis. Gene editing did not interfere with the interaction of MATα2 with AEP/PEP.

In summary, our study unveiled the functions of the secreted MATα2 in CRLM. EV-MATα2 can lower MAT1A expression, the liver’s defense to metastasis, while promoting its own oncogenic activity; the freely secreted MATα2-t is critical for cancer cell survival in part by activating FAK and promoting its own expression. Currently, there are multiple clinical trials exploring the feasibility of MAT2A inhibitors and FAK inhibitors as therapeutic options [37, 38]; however, discovery of MATα2-t and the novel application of neutralizing MATα2 antibody as a new therapeutic could possibly have wider applicability with less toxicity as it is only targeting MATα2-t, which seems to only be vital to cancer cell survival.

## Supplementary Information

Supplementary Material 1

## Data Availability

The datasets generated and/or analysed during the current study are available in the BioStudies accession number S-BSST2200.

## References

[1] Sung H, Ferlay J, Siegel RL, Laversanne M, Soerjomataram I, Jemal A, et al. Global Cancer Statistics 2020: GLOBOCAN Estimates of Incidence and Mortality Worldwide for 36 Cancers in 185 Countries. CA Cancer J Clin. 2021;71(3):209–49. 33538338 10.3322/caac.21660

[2] Siegel RL, Wagle NS, Cercek A, Smith RA, Jemal A. Colorectal cancer statistics, 2023. CA Cancer J Clin. 2023;73(3):233–54. 36856579 10.3322/caac.21772

[3] Zhou K, Yang C, Li Y. Multi-omics in colorectal cancer liver metastasis: applications and research advances. Cancer Biol Med. 2025;22(6):618–38. 40574729 10.20892/j.issn.2095-3941.2025.0066 PMC12240200

[4] Martin J, Petrillo A, Smyth EC, Shaida N, Khwaja S, Cheow HK, et al. Colorectal liver metastases: current management and future perspectives. World J Clin Oncol. 2020;11(10):761–808. 33200074 10.5306/wjco.v11.i10.761 PMC7643190

[5] Dueland S, Guren TK, Hagness M, Glimelius B, Line PD, Pfeiffer P, et al. Chemotherapy or liver transplantation for nonresectable liver metastases from colorectal cancer? Ann Surg. 2015;261(5):956–60. 24950280 10.1097/SLA.0000000000000786

[6] Tian Y, Wang Y, Wen N, Wang S, Li B, Liu G. Prognostic factors associated with early recurrence following liver resection for colorectal liver metastases: a systematic review and meta-analysis. BMC Cancer. 2024;24(1):426. 38584263 10.1186/s12885-024-12162-4 PMC11000331

[7] Su YM, Liu W, Yan XL, Wang LJ, Liu M, Wang HW, et al. Five-year survival post hepatectomy for colorectal liver metastases in a real-world Chinese cohort: recurrence patterns and prediction for potential cure. Cancer Med. 2023;12(8):9559–69. 36846977 10.1002/cam4.5732 PMC10166917

[8] Lemke J, Cammerer G, Ganser J, Scheele J, Xu P, Sander S, et al. Survival and prognostic factors of colorectal liver metastases after surgical and nonsurgical treatment. Clin Colorectal Cancer. 2016;15(4):e183–92. 27269232 10.1016/j.clcc.2016.04.007

[9] Lu SC, Mato JM. S-adenosylmethionine in liver health, injury, and cancer. Physiol Rev. 2012;92(4):1515–42. 23073625 10.1152/physrev.00047.2011 PMC3698976

[10] Murray B, Barbier-Torres L, Fan W, Mato JM, Lu SC. Methionine adenosyltransferases in liver cancer. World J Gastroenterol. 2019;25(31):4300–19. 31496615 10.3748/wjg.v25.i31.4300 PMC6710175

[11] Lu L, Zhang J, Fan W, Li Y, Wang J, Li TWH, et al. Deregulated 14-3-3zeta and methionine adenosyltransferase alpha1 interplay promotes liver cancer tumorigenesis in mice and humans. Oncogene. 2021;40(39):5866–79. 34349244 10.1038/s41388-021-01980-6 PMC9611740

[12] Yang H, Huang ZZ, Zeng Z, Chen C, Selby RR, Lu SC. Role of promoter methylation in increased methionine adenosyltransferase 2A expression in human liver cancer. Am J Physiol Gastrointest Liver Physiol. 2001;280(2):G184–90. 11208539 10.1152/ajpgi.2001.280.2.G184

[13] Chen H, Xia M, Lin M, Yang H, Kuhlenkamp J, Li T, et al. Role of methionine adenosyltransferase 2A and S-adenosylmethionine in mitogen-induced growth of human colon cancer cells. Gastroenterology. 2007;133(1):207–18. 17631143 10.1053/j.gastro.2007.03.114

[14] Wang X, Guo X, Yu W, Li C, Gui Y, Cai Z. Expression of methionine adenosyltransferase 2A in renal cell carcinomas and potential mechanism for kidney carcinogenesis. BMC Cancer. 2014;14:196. 24636201 10.1186/1471-2407-14-196 PMC4003826

[15] Tomasi ML, Ryoo M, Ramani K, Tomasi I, Giordano P, Mato JM, et al. <article-title update="added">Methionine adenosyltransferase α2 sumoylation positively regulate Bcl-2 expression in human colon and liver cancer cells. Oncotarget. 2015;6(35):37706–23. 26416353 10.18632/oncotarget.5342 PMC4741959

[16] Maldonado LY, Arsene D, Mato JM, Lu SC. Methionine adenosyltransferases in cancers: mechanisms of dysregulation and implications for therapy. Exp Biol Med (Maywood). 2018;243(2):107–17. 29141455 10.1177/1535370217740860 PMC5788143

[17] Secker KA, Bloechl B, Keppeler H, Duerr-Stoerzer S, Schmid H, Schneidawind D, et al. MAT2A as key regulator and therapeutic target in MLLr leukemogenesis. Cancers (Basel). 2020. 10.3390/cancers12051342. 32456310 10.3390/cancers12051342 PMC7281730

[18] Chu PY, Wu HJ, Wang SM, Chen PM, Tang FY, Chiang EI. MAT2A localization and its independently prognostic relevance in breast cancer patients. Int J Mol Sci. 2021;22(10)5382-96 10.3390/ijms22105382 PMC8161225 34065390

[19] Fan W, Cao D, Yang B, Wang J, Li X, Kitka D, et al. Hepatic prohibitin 1 and methionine adenosyltransferase alpha1 defend against primary and secondary liver cancer metastasis. J Hepatol. 2024;80(3):443–53. 38086446 10.1016/j.jhep.2023.11.022 PMC10922446

[20] Kim HY, Lee W, Liu X, Jang H, Sakane S, Carvalho-Gontijo Weber R, et al. Protocol to generate human liver spheroids to study liver fibrosis induced by metabolic stress. STAR Protoc. 2024;5(2):103111. 38833372 10.1016/j.xpro.2024.103111 PMC11179098

[21] Worsley Hunt R, Wasserman WW. Non-targeted transcription factors motifs are a systemic component of ChIP-seq datasets. Genome Biol. 2014;15(7):412. 25070602 10.1186/s13059-014-0412-4 PMC4165360

[22] Mao Z, Liu S, Cai J, Huang ZZ, Lu SC. Cloning and functional characterization of the 5’-flanking region of human methionine adenosyltransferase 2A gene. Biochem Biophys Res Commun. 1998;248(3):479–84. 9703951 10.1006/bbrc.1998.8965

[23] Zeng Z, Huang ZZ, Chen C, Yang H, Mao Z, Lu SC. Cloning and functional characterization of the 5’-flanking region of human methionine adenosyltransferase 1A gene. Biochem J. 2000;346:475–82. 10677369 PMC1220876

[24] Ott JM, Gassenmaier V, Bitzer M, Schurch CM, Heitmann JS, Hagelstein I. B7-H3: a consistent marker in metastatic colorectal cancer with potential for targeted treatment. Pathol Oncol Res. 2025;31:1612186. 40881578 10.3389/pore.2025.1612186 PMC12380622

[25] Ramani K, Lu SC. Methionine adenosyltransferases in liver health and diseases. Liver Res. 2017;1(2):103–11. 29170720 10.1016/j.livres.2017.07.002 PMC5695885

[26] Tan X, Yan Y, Song B, Zhu S, Mei Q, Wu K. Focal adhesion kinase: from biological functions to therapeutic strategies. Exp Hematol Oncol. 2023;12(1):83. 37749625 10.1186/s40164-023-00446-7 PMC10519103

[27] Zhang W, Lin Y. The mechanism of asparagine endopeptidase in the progression of malignant tumors: a review. Cells. 2021. 10.3390/cells10051153. 34068767 10.3390/cells10051153 PMC8151911

[28] Perez RE, Calhoun S, Shim D, Levenson VV, Duan L, Maki CG. Prolyl endopeptidase inhibitor Y-29794 blocks the IRS1-AKT-mTORC1 pathway and inhibits survival and in vivo tumor growth of triple-negative breast cancer. Cancer Biol Ther. 2020;21(11):1033–40. 33044914 10.1080/15384047.2020.1824989 PMC7678932

[29] Xu W, Xu J, Liu J, Wang N, Zhou L, Guo J. Liver metastasis in cancer: molecular mechanisms and management. MedComm. 2025;6(3):e70119. 40027151 10.1002/mco2.70119 PMC11868442

[30] Zhou H, Liu Z, Wang Y, Wen X, Amador EH, Yuan L, et al. Colorectal liver metastasis: molecular mechanism and interventional therapy. Signal Transduct Target Ther. 2022;7(1):70. 35246503 10.1038/s41392-022-00922-2 PMC8897452

[31] Andryszkiewicz W, Misiag P, Karwowska A, Resler Z, Wojno A, Kulbacka J et al. Cancer metastases to the liver: mechanisms of tumor cell colonization. Pharmaceuticals (Basel). 2024;17(9). 10.3390/ph17091251 PMC11434846 39338413

[32] Wang Z, Kim SY, Tu W, Kim J, Xu A, Yang YM, et al. Extracellular vesicles in fatty liver promote a metastatic tumor microenvironment. Cell Metab. 2023;35(7):1209–e2613. 37172577 10.1016/j.cmet.2023.04.013 PMC10524732

[33] Tomasi ML, Ryoo M, Skay A, Tomasi I, Giordano P, Mato JM, et al. Polyamine and methionine adenosyltransferase 2A crosstalk in human colon and liver cancer. Exp Cell Res. 2013;319(12):1902–11. 23588207 10.1016/j.yexcr.2013.04.005 PMC3700574

[34] Yu G, Xu M, Zhou L, Zheng K, Zhu X, Sui J, et al. High expression of phosphorylated focal adhesion kinase predicts a poor prognosis in human colorectal cancer. Front Pharmacol. 2022;13:989999. 36176444 10.3389/fphar.2022.989999 PMC9513477

[35] Pañeda C, Gorospe I, Herrera B, Nakamura T, Fabregat I, Varela-Nieto I. Liver cell proliferation requires methionine adenosyltransferase 2A mRNA up-regulation. Hepatology. 2002;35(6):1381–91. 12029623 10.1053/jhep.2002.32538

[36] Morillo-Huesca M, I GL-C, Conesa-Bakkali R, Tome M, Watts C, Huertas P, et al. Radiotherapy resistance driven by asparagine endopeptidase through ATR pathway modulation in breast cancer. J Exp Clin Cancer Res. 2025;44(1):74. 40012043 10.1186/s13046-025-03334-6 PMC11866873

[37] Jeong KY. Inhibiting focal adhesion kinase: a potential target for enhancing therapeutic efficacy in colorectal cancer therapy. World J Gastrointest Oncol. 2018;10(10):290–2. 30364839 10.4251/wjgo.v10.i10.290 PMC6198301

[38] Pulous FE, Steurer B, Pun FW, Zhang M, Ren F, Zhavoronkov A. MAT2A inhibition combats metabolic and transcriptional reprogramming in cancer. Drug Discov Today. 2024;29(11):104189. 39306235 10.1016/j.drudis.2024.104189

