# Supplementary material for: A novel role of secreted methionine adenosyltransferase α2 in colorectal liver metastases
Source: J Exp Clin Cancer Res. 2025 Dec 2;45:8. doi: 10.1186/s13046-025-03599-x (PMC12777475; doi:10.1186/s13046-025-03599-x)
Supplement: Supplementary file 1 — Supplementary Material 1 [file 13046_2025_3599_MOESM1_ESM.docx]

**SUPPLEMENTARY MATERIAL**

**Supplemental Figure 1. MATα2 binding to human *MAT1A* and *MAT2A* promoter regions.** Binding sites were predicted based on motif discovery analysis of ChIP-seq signal enrichment across the promoter regions. **(A)** ChIP-seq analysis reveals MATα2 binding within the human *MAT1A* promoter. Genomic binding coordinates (hg19) are shown at the top, with MATα2 binding regions highlighted in red. The corresponding *MAT1A* promoter sequence is displayed, with predicted MATα2 binding motifs highlighted in color: yellow (AATGC), cyan (AATGCAA), green (AATGATC), pink (AATGCC), dark green (AATGAATC) and grey (AATGGAC). **(B)** ChIP-seq analysis of the human *MAT2A* promoter identifies MATα2 binding enrichment. Genomic coordinates (hg19) are shown at the top, with MATα2 binding regions depicted in blue. The *MAT2A* promoter sequence is presented with candidate MATα2 binding motifs highlighted in color: yellow (AAAT), grey (AAATGGAC/AATCTGC) and cyan (AATCCCC). TATA box is indicated in red color.

**Figure 2. EV-MATα2 binds to *MAT2A* promoter in HT29 cells.** HT29 cells were treated with exosomes from HT29 cells expressing empty vector (EVec exo) or MAT2A-His-Tag vector (EV-MATα2) as described in Methods. ChIP analysis analyzed the binding of MATα2-His to different predicted motifs in human *MAT2A* promoter. Purified genomic DNA (Input) is used as normalization control, while IgG negative control for non-specific antibody binding (M: marker). Mean ± SEM from n=3, **p*< 0.05 vs. EVec exo.

**Figure 3. Verification of the N-terminal cleavage of secreted MATα2.** RKO cells were transfected with empty vector (EVec) or DDK-MAT2A-His-Tag construct for 48 hours. Cell lysate and conditioned media were analyzed by SDS-PAGE. Membrane was blotted for DDK and His. Actin was used as loading control.

**Supplemental Figure 4. MATα2 is highly secreted in HT29 cells.** Comparison of MATα2 secretion in normal human colon epithelial (HCoEpC) and HT29 cells. Western blot of culture media (left) and cell lysates (right) show elevated levels of MATα2 and MATα2-t in HT29 cells compared to HCoEpC cells. Ponceau and actin were used as loading controls. Mean ± SEM from n=3, *p<0.04 and †p<0.01 vs. HCoEpC.

**Supplemental Figure 5. Functional effects of secreted MATα2. (A)** (left) Human PBMCs were isolated as described in Methods and treated with anti-MATα2 Ab for 48 hours and western blotted for MATα2, pro-casp3 and active casp3 (undetectable). Cell lysates (Ly), lysates plus anti-MATα2 antibody (Ly+Ab), culture media (Med), or culture media plus antibody (Med+Ab). Actin was used as loading control. (Right) TUNEL staining of HCoEpC shows no increase in apoptosis upon treatment with anti-MATα2 antibody compared to control. **(B)** Immunoblotting of HT29 cells treated with control or anti-MATα2 antibody to detect phosphorylated FAK (pFAK), total FAK, pro-casp3, active casp3 and actin. Mean ± SEM from n=3, *p<0.04 and †p<0.05 vs. control. **(C)** Western blot analysis of secreted truncated MATα2 (MATα2-t) in culture media from wild-type (WT) and gene edited HT29 cells at the PDLD motif (HDR). Ponceau staining was used as control loading. Mean ± SEM from n=3, *p<0.05 vs WT.

**Supplemental Figure 6. MATα2 interacts with PEP and AEP in CRC cells. (A)** RKO and HT29 cells were transfected with empty vector (EVec) or *MAT2A* overexpression vector and co-immunoprecipitated with anti-MATα2 Ab. Next, membranes were blotted for PEP and AEP. IgG were used as a negative control and actin a loading control. Mean ± SEM from n=3, *p<0.001 vs EVec for RKO cells; *p<0.04 and †p<0.05 vs EVec for HT29 cells. **(B)** RKO cells were CRISPR/HDR-edited cells at proline 30 as described in Methods, processed for co-IP with anti-MATα2 Ab and blotted for MATα2, PEP and AEP. Mean ± SEM from n=3.


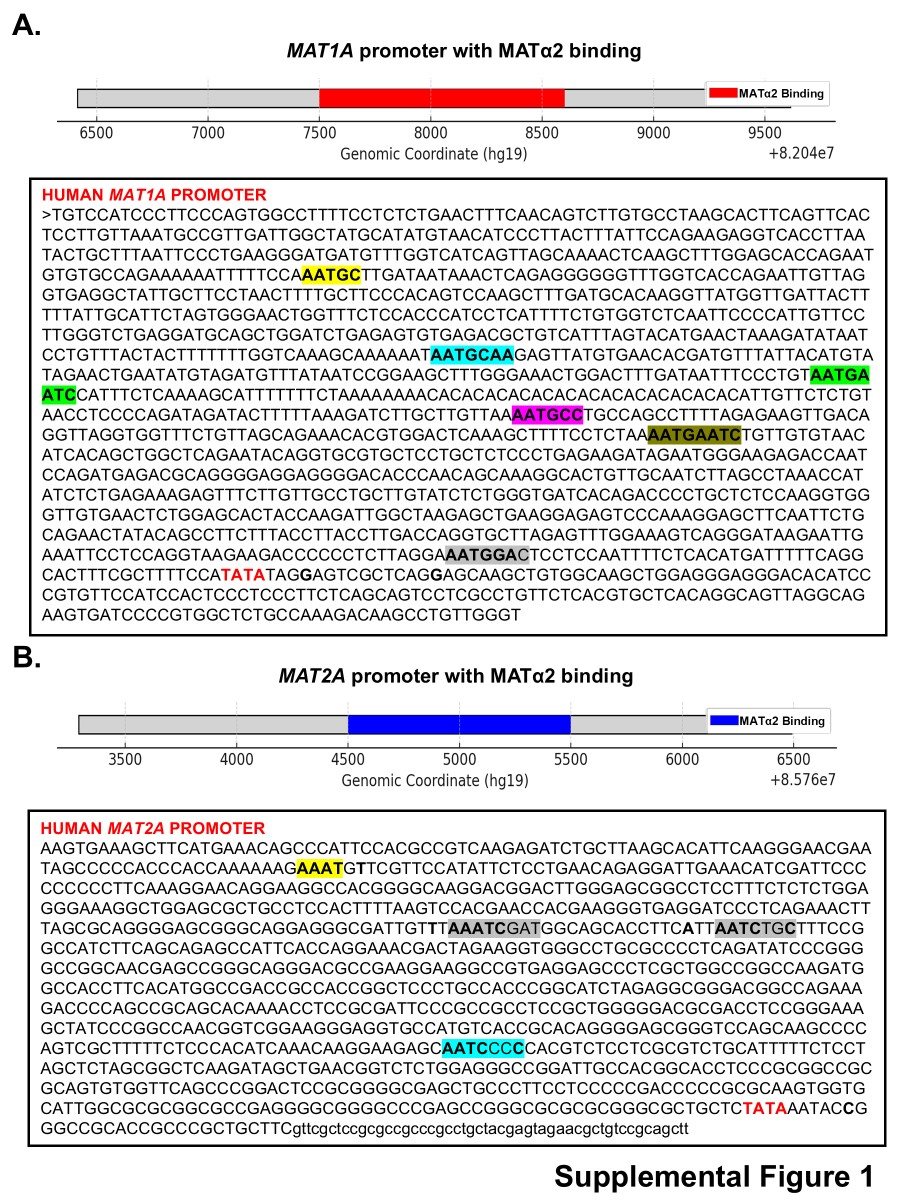


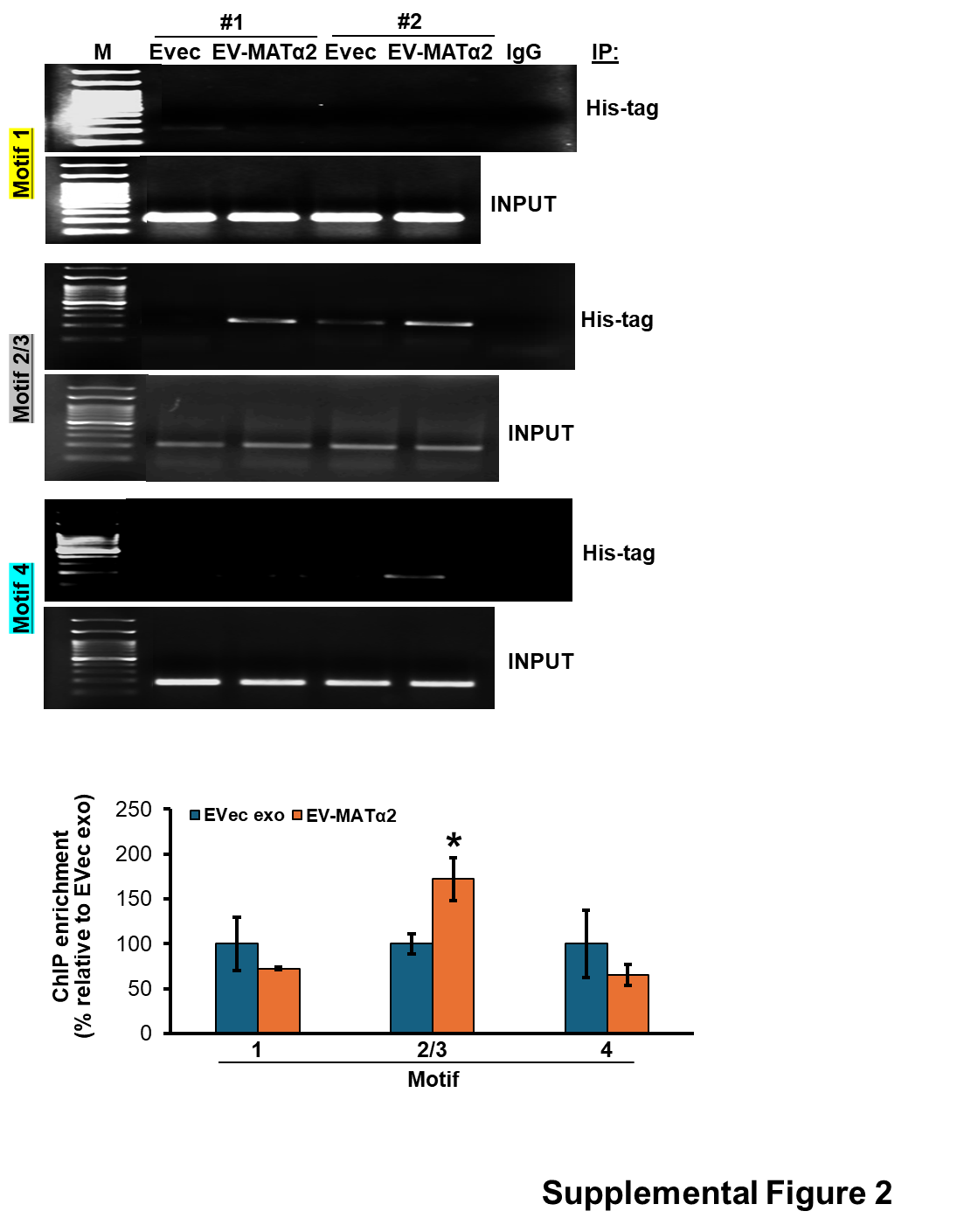


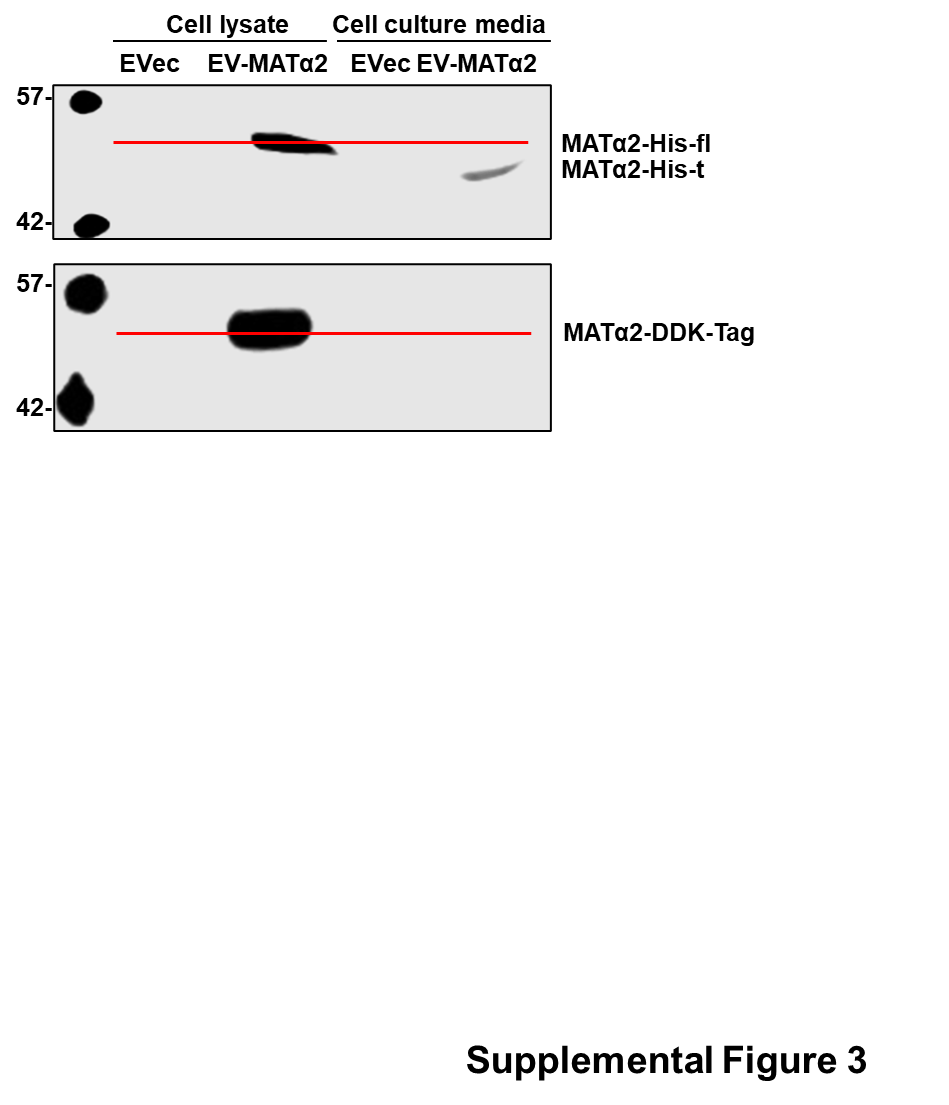


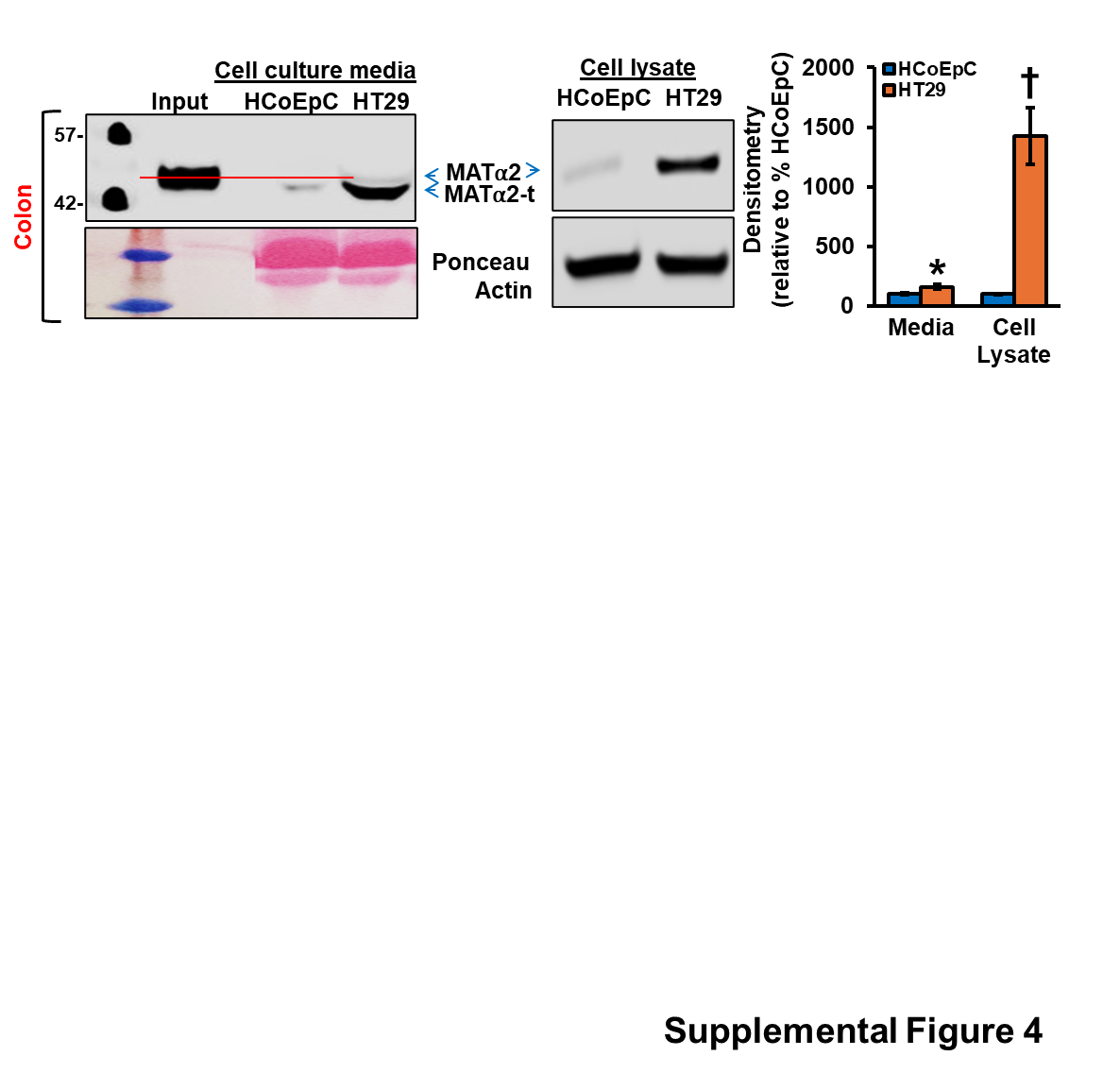


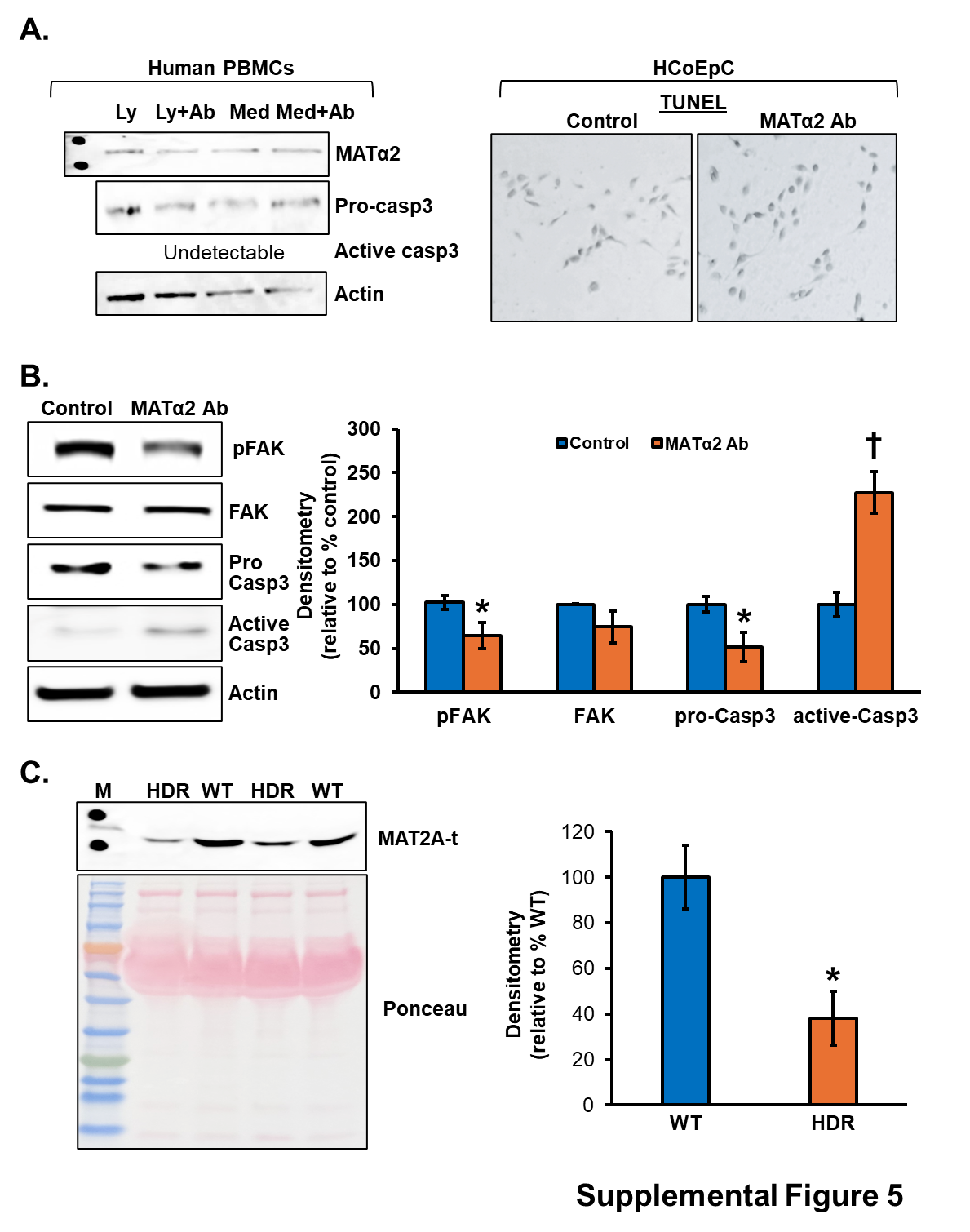


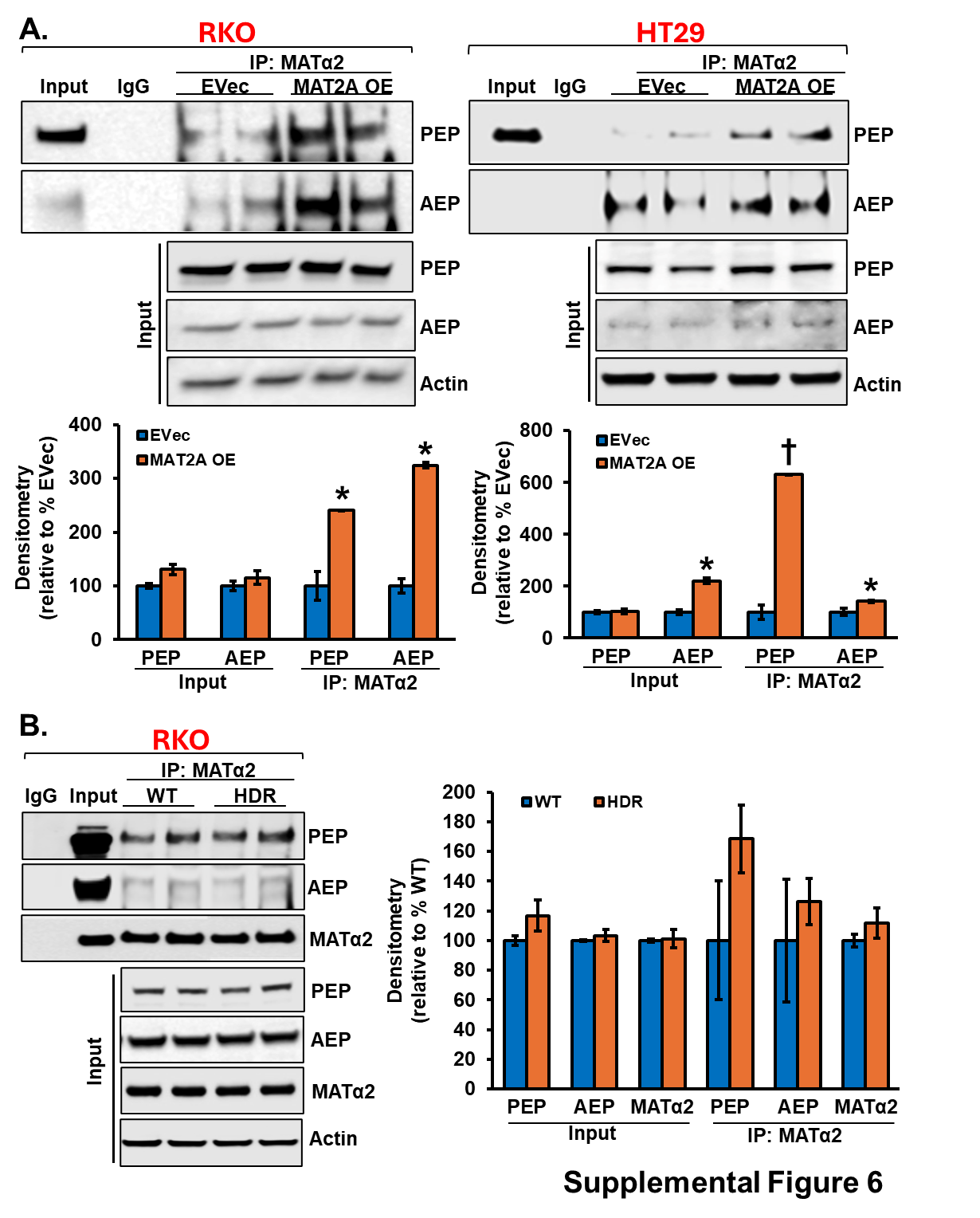


**Supplemental Table 1: List of antibodies.**

| **Antibodies** | **Application** | **Catalog #** | **Manufacturer** |
| --- | --- | --- | --- |
| **MATα1** | IF | PA5-115549 | Invitrogen |
|  | IHC | H0004143-M01 | Abnova |
| **MAT II Antibody (F-12) AC** | IP | sc-398917 AC | Santa Cruz |
| **MATα2** | WB | NBP-92100 | Novus |
|  | IHC | NB110-94158 | Novus |
| **Anti-DDK** | WB, IF | 8146S | Cell Signaling |
| **Anti-His** | IP, IF, ChIP | 66005-1-Ig | Proteintech |
| **FAK** | WB | GTX100764 | Genetex |
| **pFAK** | WB | 3281S | Cell Signaling |
| **AEP** | WB | 67017-1-Ig | Proteintech |
| **PEP** | WB | 11536-1-AP | Proteintech |
| **Caspase-3** | WB | sc-56053 | Santa Cruz |
| **MATα2** | WB | ab206386 | Abcam |
| **MATα2** | WB, IF | sc-398917 | Santa Cruz |
| **HSP70** | WB | 10995-1-AP | Proteintech |
| **CD9** | WB | 20597-1-AP | Proteintech |
| **Lamin B1** | WB | 12987-1-AP | Proteintech |
| **GAPDH** | WB | GTX627408 | Genetex |
| **Tubulin** | WB | HRP-66031 | Proteintech |
| **Anti-β-Actin−Peroxidase** | WB | A3854-200UL | Sigma |
| **Rabbit Alexa fluor 594** | IF | ab150080 | Abcam |
| **Mouse Alexa fluor 488** | IF | ab150117 | Abcam |
| **Mouse Alexa fluor 594** | IF | ab150116 | Abcam |
| **Anti-rabbit IgG HRP-linked** | WB | 7074S | Cell Signaling |
| **Anti-mouse IgG HRP-linked** | WB | 7076S | Cell Signaling |
| **Veriblot** | IP | ab131366 | Abcam |
| **POL II** | ChIP | sc-13583 | Santa Cruz |
| **Normal IgG** | IP | sc69786 | Santa Cruz |

**Supplemental Table 2: List of probes (qRT-PCR) and primers (ChIP).**

| **qRT-PCR Probes** | | | |
| --- | --- | --- | --- |
| **Gene** | | **Catalog #** | **Supplier** |
| *Mat2a* | | Hs00428515_g1 | Thermo Fisher |
| *Mat1a* | | Hs01547962_m1 |  |
| *Ptk2* | | Hs01056457_m1 |  |
| *Hprt1* | | Hs99999909_m1 |  |
| **ChIP Primers** | | | |
| **Promoter** | **Motif** | **Sequence (5’- 3’)** | |
|  |  | **Forward** | **Reverse** |
| *Mat1a* | Motif#1 | CCTCTCTGAACTTTCAACAG | CCAGTTCCCACTAGAATGC |
|  | Motif #2 | GCATTCTAGTGGGAACTGG | AGTCCAGTTTCCCAAAGCTTCC |
|  | Motif #3 | AATCCGGAAGCTTTGGGAAA | CTATCTGGGGAGGTTACAG |
|  | Motif #4 | CACACACACATTGTTCTCTG | CTTTGAGTCCACGTGTTTCT |
|  | Motif #5 | AGAGAAGTTGACAGGTTAGG | GTGCTCCAGAGTTCACAAC |
|  | Motif #6 | GTTGTGAACTCTGGAGCAC | TGAGCGACTCCTATATATGG |
| *Mat2a* | Motif#1 | GCTTCATGAAACAGCCCATTCCA | CTTTCCCTCCAGAGAGAAAGGA |
|  | Motif #2/3 | CGTTTCCTGGTGAATGGCTCT | TCAAAGGAACAGGAAGGCCAC |
|  | Motif #4 | CCAGTCGCTTTTTCTCCCACAT | AGTCCGGGCTGAACCACA |
